# Machine Learning-Guided Synthetic Microbial Communities Enable Functional and Sustainable Degradation of Persistent Environmental Pollutants

**DOI:** 10.1021/acs.est.6c01112

**Published:** 2026-04-27

**Authors:** Esaú De la Vega-Camarillo, Jorge Arreola-Vargas, Sanjay Antony-Babu, Saurav Kumar Mathur, Joshua Andrew Santos, Won Bo Shim

**Affiliations:** Department of Plant Pathology and Microbiology, 14736 Texas A&M University, College Station, Texas 77843, United States

**Keywords:** PFAS biodegradation, synthetic microbial communities, machine learning, graph neural networks, atrazine, lignin, metabolic complementarity, whole genome sequencing, bioremediation

## Abstract

Persistent environmental pollutants require diverse microbial metabolic capabilities for effective degradation. While naturally occurring consortia or single strains often fall short in efficiency, synthetic microbial communities (SynComs) hold greater promise for enhanced degradation. To address this challenge, we developed GENIA (Genomically and Environmentally Networked Intelligent Assemblies), a genome-informed, machine learning-guided framework for the rational design of SynComs capable of degrading multiple pollutants. Using a microfluidic high-throughput cultivation platform, 2,155 bacterial strains were isolated from xenobiotic-enriched cotton detritusphere and screened for pollutant-specific growth. Whole-genome sequencing and functional annotation of 45 prioritized strains revealed metabolic traits associated with the degradation of lignin, atrazine, and PFAS. These genomic profiles were encoded into spline-based graph representations and integrated within the GENIA pipeline, which combines graph neural networks, pathway complementarity modeling, and functional redundancy minimization to predict optimal community assemblies. The resulting nine-member community, comprising *Atlantibacter hermannii*, *Bacillus cabrialesii*, *Bacillus licheniformis*, *Bacillus pseudomycoides*, *Micrococcus luteus*, *Paenibacillus polymyxa*, *Pantoea dispersa*, *Pseudomonas fulva*, and *Pseudomonas pergaminensis*, demonstrated broad catabolic capacity. Kinetic experiments in minimal medium showed simultaneous multipollutant degradation: lignin (91.6% by day 5), atrazine (91.4% by day 3), and PFOS (93.1% within 7 days), representing 2.2-fold improvement over best individual performers. Full-length 16S rRNA metabarcoding confirmed stable community composition with predicted hub strains expanding to 14–15.6% relative abundance. Soil microcosm validation demonstrated >70% degradation at 3 weeks. GENIA establishes a scalable framework that integrates systems genomics, phenotypic screening, and predictive modeling to engineer microbial consortia for complex environmental bioremediation.

## Graphical abstract

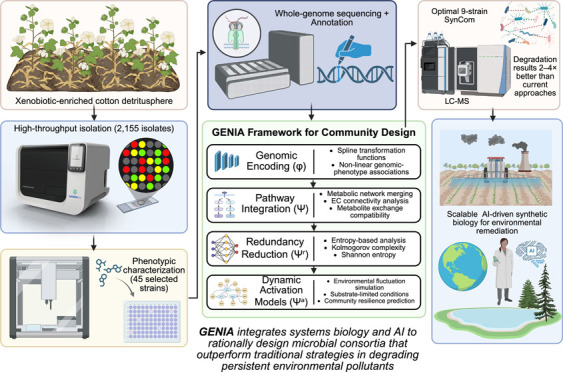

## Introduction

Microbial communities have shaped life on Earth not through competition, but through cooperation, a principle captured by Lynn Margulis: *″Life did not take over the globe by combat, but by networking″*.^1^ This ecological interdependence inspires synthetic microbial communities (SynComs), intentionally assembled to perform functions beyond the reach of individual strains or natural consortia. Despite its conceptual appeal, few studies have validated SynCom as a practical strategy for solving real-world environmental problems. Persistent pollutants such as lignin, atrazine, and per- and polyfluoroalkyl substances (PFAS) exemplify global environmental challenges that demand innovative remediation approaches.^2–5^ These pollutants pose persistent threats to both terrestrial and aquatic ecosystems due to their structural stability and widespread use in agriculture and industry.^6–8^ Atrazine, a widely used herbicide, persists in groundwater and has been associated with endocrine disruption and ecotoxicity.^9–11^ Lignin, although natural, is a complex aromatic polymer that hinders biomass valorization and requires microbial degradation for effective bioconversion.^12,13^ PFAS, including perfluorooctanoic acid (PFOA) and perfluorooctanesulfonate (PFOS), are highly fluorinated compounds used in coatings and surfactants that are resistant to degradation, leading to global contamination and bioaccumulation.^14–16^

Microbial bioremediation has been explored as a sustainable strategy for degrading these pollutants, leveraging enzymes such as peroxidases, dehalogenases, and monooxygenases.^17–19^ However, most natural microbial consortia exhibit functional redundancy and competitive interactions that limit degradation efficiency.^20^ Furthermore, the isolation and characterization of novel strains with xenobiotic-degrading capabilities remain a bottleneck, particularly for pollutants with low bioavailability or limited membrane transport.^21^

To address these limitations, high-throughput microfluidic platforms, such as the Prospector system, enable the cultivation of challenging or slow-growing environmental bacteria by enabling nanowell-scale isolation, minimizing interspecies competition, and promoting unbiased recovery of environmental taxa.^22,23^ Following isolation, genome-resolved metabolic modeling can guide rational community design by integrating genomic potential with functional performance. Recent advances in whole-genome sequencing, particularly using long-read platforms such as Oxford Nanopore Technologies, have enhanced the resolution of biosynthetic and degradative pathways in environmental strains.^24–26^

In parallel, machine learning (ML) and network-based modeling frameworks have shown promise in designing SynComs for targeted functions, including biodegradation and nutrient cycling.^27,28^ Graph Neural Networks (GNNs) and attention-based architectures enable integration of heterogeneous biological data, allowing predictions of emergent properties such as community stability and pollutant degradation capacity.^29–31^ However, these computational approaches are often developed independently of experimental validation, limiting their practical utility.

Here, we present an integrated experimental-computational framework, GENIA (Genomically and Environmentally Networked Intelligent Assemblies), that couples high-throughput strain isolation, functional screening, genome sequencing, and network modeling to design synthetic communities for the degradation of lignin, atrazine, and PFAS. We isolated 2,155 bacterial strains from the cotton detritusphere, a complex, microbially active niche shaped by intensive agrochemical use and enriched in recalcitrant compounds such as lignin (from plant biomass), atrazine (a legacy herbicide), and per- and polyfluoroalkyl substances (PFAS) from irrigation and soil amendments. We performed systematic phenotypic screening on defined pollutant substrates. Genomes of the most effective degraders (n = 45) were sequenced, annotated, and analyzed for key catabolic enzymes and transporters.

Using spline-based genome encodings and metabolic network integration, we constructed predictive models of community-level degradation. Functional redundancy was minimized using Kolmogorov complexity analysis, and dynamic activation models were used to simulate pathway engagement under stress. Community performance was predicted using a hybrid GNN architecture incorporating Graph Attention Networks and Node2Vec embeddings, trained on empirical growth data.

This study presents the first demonstration of machine-learning-guided synthetic community assembly for the simultaneous degradation of structurally diverse persistent pollutants, establishing a comprehensive workflow that integrates high-throughput microbiology, systems genomics, and predictive AI modeling.

## Materials and Methods

### Overall Study Design

The strategy encompassed field, laboratory, informatics, and in vitro evaluations to develop and validate a minimal synthetic community capable of degrading targeted environmental pollutants, including PFAS, atrazine, and lignin. First, we recovered a large number of bacterial isolates using the Prospector high-throughput culturomics method, enabling us to screen for bacteria that can utilize pollutants of interest as a nutritional substrate and potentially degrade them. The bacteria identified as potential degraders were sequenced, and our novel GENIA strategy (Figure 1) was applied to genome-scale models. Using a set of machine learning strategies, we identified low-redundancy and complementary communities that can efficiently degrade all three pollutants of interest. Finally, we validated degradation performance in liquid culture and soil microcosms. Detailed descriptions are presented below.

**Figure 1:**
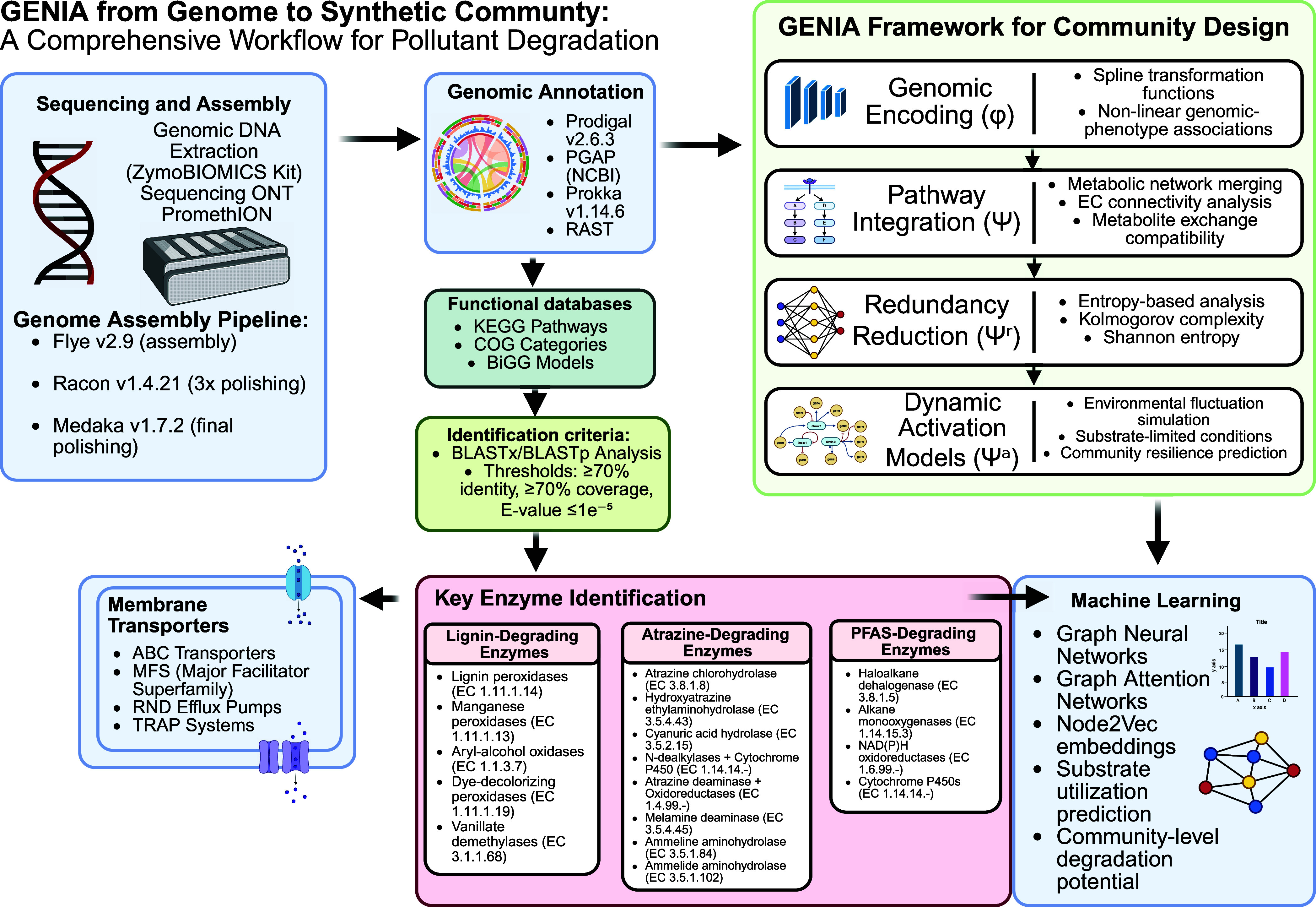
GENIA framework for machine learning-guided design of pollutant-degrading synthetic microbial communities. The integrated workflow combines high-throughput isolation, Oxford nanopore sequencing, and comprehensive annotation (Prodigal, PGAP, Prokka, RAST) with KEGG, COG, and BiGG databases. GENIA processes genomic data through four sequential modules: (1) Genomic encoding (φ) using spline transformations, (2) pathway Integration (Ψ) for metabolic network merging, (3) redundancy reduction (Ψ^r^) employing Kolmogorov complexity analysis, and (4) dynamic activation models (Ψ^a^) for environmental simulation. Machine learning integration uses graph neural networks and Node2Vec embeddings to predict optimal synthetic communities targeting lignin, atrazine, and PFAS degradation pathways with enhanced capacity and minimal competition.

### High-Throughput Culturomics Using Prospector System

Bacteria were isolated from cotton stalk-associated detritusphere using the Prospector Microbial Isolation and Cultivation System (Isolation Bio, San Carlos, CA). The detritusphere was selected as an isolation source due to its enrichment in lignocellulosic residues and a legacy of agricultural and industrial contaminants, including atrazine and per- and polyfluoroalkyl substances (PFAS).^32,33^ The cotton stalks were collected from Texas A&M Brazos Bottom Experimental Farm Station (30.551157826632647, −96.43135829477686). These stalks had been left behind after harvest in November 2024 and were sampled for our study in April 2025. For microbial extraction, 0.5 g of lignocellulosic material was weighed, diluted with 4.5 mL of PBS, and vortexed for 1 min to dislodge large soil particles. Samples were then sonicated for 5 min at 40 kHz frequency to dislodge microorganisms from the detritusphere. Serial dilutions of this suspension were prepared in R2A medium using the Most Probable Number (MPN) technique with OzBlue as a redox indicator for growth detection. MPN-based quantification of the original microbial concentration was subsequently used to calculate the dilution factor required to achieve a target density of approximately one cell per 3 nL.^34^ Diluted suspensions (1.5 mL) were mixed with 1.5 mL R2A medium containing the Prospector viability dye (final concentration: 200 μmol L^–1^) and loaded into arrays of >6000 nanowells (3 nL per well) using the system’s vacuum-loading chamber. Arrays were sealed and incubated aerobically at 27 °C for 48 h. Viable isolates were identified based on fluorescence changes (excitation: 530 nm; emission: 590 nm) and aseptically transferred to 96-well plates containing R2A broth using the automated Prospector transfer system.^35^ A total of 2,155 pure isolates were obtained.

### Functional Screening of Pollutant Degradation Potential

To assess the potential for pollutant degradation, isolates were screened for growth using lignin, atrazine, or PFAS as primary carbon sources in minimal medium. Analytical-grade compounds were used throughout: alkali lignin (Sigma-Aldrich, ≥ 97% purity), atrazine (Tokyo Chemical Industry Co., > 97% purity), perfluorooctanesulfonic acid potassium salt (PFOS-K, Agilent Technologies, ≥ 98% purity), and perfluorooctanoic acid (PFOA, Agilent Technologies, ≥ 95% purity). PFAS compounds were supplied as stock solutions in methanol and added to achieve final concentrations of 20 mg L^–1^ each, with the methanol content maintained below 0.04% (v/v). Control treatments with equivalent methanol concentrations without PFAS were included to account for potential solvent effects. Before inoculation, cells were washed twice with phosphate-buffered saline (PBS, pH 7.0), resuspended, and normalized to an optical density (OD_600_) of 0.5. Automated inoculation into 96-well plates was performed using the Opentrons OT-2 liquid handling platform.^36^ Each well received 20 μL of normalized inoculum, 180 μL of M9 minimal medium (Na_2_HPO_2_, 6.78 g L^–1^; KH_2_PO_4_, 3.0 g L^–1^; NH_4_Cl, 1.0 g L^–1^; NaCl, 0.5 g L^–1^; MgSO_4_, 1 mM; CaCl_2_, 0.1 mM), supplemented with alkali lignin (500 mg L^–1^), atrazine (30 mg L^–1^), or PFAS (20 mg L^–1^ each of PFOA and PFOS)^13,37,38^ and 5 μL OzBlue redox indicator. Cultures were incubated at 27 °C for 10 days with orbital shaking at 120 rpm. Growth was assessed daily by measuring relative fluorescence units (RFU) at 560 nm excitation and 590 nm emission wavelengths using a microplate reader (SpectraMax, Molecular Devices). Isolates were considered positive for pollutant utilization when fluorescence readings exceeded the mean fluorescence of negative controls by at least 2 standard deviations and exhibited sustained growth for at least 3 consecutive days. Forty-five strains demonstrating consistent growth on at least one pollutant were selected for further analysis.

### Whole-Genome Sequencing and Assembly

Genomic DNA was extracted from each isolate using the ZymoBIOMICS DNA Miniprep Kit (Zymo Research, USA). High-molecular-weight DNA was used to construct sequencing libraries using Oxford Nanopore standard protocols. Sequencing was performed on the PromethION platform (Plasmidsaurus, Eugene, OR). Genome assemblies were generated using Flye v2.9,^39^ followed by three rounds of polishing with Racon v1.4.21 ^40^ and one round with Medaka v1.7.2.^41^ Assembly quality was assessed using QUAST v5.2.0,^42^ and completeness and contamination were evaluated with CheckM v1.2.2,^43^ ensuring >98% completeness and <1.5% contamination. All genomes deposited in NCBI GenBank under BioProject PRJNA1370521.

### Genome Annotation and Pathway Characterization

Genome annotation was performed using PGAP,^44^ Prokka v1.14.6,^45^ and RAST.^46^ Prodigal v2.6.3 was used for gene prediction.^47^ Functional annotation employed KEGG,^48^ COG,^49^ and BiGG Models database.^50^ BLASTx and BLASTp were used to identify orthologs relevant to biodegradation with thresholds of ≥ 70% identity, ≥ 70% query coverage, and E-value ≤ 1e^–5^.^51^ Specific enzymes were identified and classified by their EC numbers using functional annotation databases. Lignin-degrading enzymes included lignin peroxidases (EC 1.11.1.14), manganese peroxidases (EC 1.11.1.13), aryl-alcohol oxidases (EC 1.1.3.7), dye-decolorizing peroxidases (EC 1.11.1.19), and vanillate demethylases (EC 3.1.1.68). Atrazine degradation pathways included atrazine chlorohydrolase (EC 3.8.1.8), hydroxyatrazine ethylaminohydrolase (EC 3.5.4.43), and cyanuric acid hydrolase (EC 3.5.2.15); the dealkylation pathway involving N-dealkylases and cytochrome P450 enzymes (EC 1.14.14.-); and the oxidative pathway with atrazine deaminase and various oxidoreductases (EC 1.4.99.-). Additional enzymes included melamine deaminase (EC 3.5.4.45), ammeline aminohydrolase (EC 3.5.1.84), and ammelide aminohydrolase (EC 3.5.1.102) for complete triazine ring mineralization. For PFAS, enzymes such as haloalkane dehalogenase (EC 3.8.1.5), alkane monooxygenases (EC 1.14.15.3), NAD­(P)H oxidoreductases (EC 1.6.99.-), and cytochrome P450s (EC 1.14.14.-) were detected. Genes encoding membrane transporters, including ABC transporters, MFS, RND efflux pumps, and TRAP systems, were frequently associated with atrazine and PFAS metabolism.

### Community Design and Network Integration Modeling (GENIA Framework)

The GENIA framework shown in Figure 1 guided the design of the synthetic community. Annotated genomes encoded using B-spline transformation functions (φ) (degree = 3, knots = 10), capturing nonlinear gene-phenotype relationships,^52^ yielding 534 spline coefficients from 267 enzyme counts. Node features augmented with 128-dimensional Node2Vec embeddings (walk length = 80, walks = 10, context = 10, p = 1, q = 0.5), producing 662-dimensional representations. Encoded profiles were processed by the pathway integration module (Ψ), which generated composite networks by merging individual strain metabolic maps based on EC connectivity and metabolite exchange compatibility.^53–55^

Graph structure: 350 nodes (45 strains, 267 enzymes, 38 metabolites), 1,247 edges (strain-enzyme binary, enzyme–substrate weighted by k_cat, strain–strain metabolic complementarity). Graph Attention Network: 3 layers with 8-head attention, dimensions [534 to 256 to 128 to 64], ELU activation, global attention pooling. MLP prediction head [64 to 32 to 16 to 3] with sigmoid output for degradation efficiency y∈[0,1]^3^.^60–62^

Training: Adam optimizer (η = 0.001), combined loss L = MSE+λ_1_L_reg+λ_2_L_stability (λ_1_ = 0.001, λ_2_ = 0.01), dropout p = 0.3, batch normalization, cosine annealing. Leave-one-out cross-validation yielded R^2^=0.763, RMSE = 0.087, Spearman ρ = 0.832 (*p* < 0.001). Redundancy reduction via Kolmogorov complexity, dynamic activation modeling via constraint-based methods.^56,57^ Community selection by genetic algorithm maximizing degradation while minimizing redundancy (k = 9, γ = 0.15). Community resilience and conditional functionality were predicted using dynamic activation models (Ψ^a^) that simulated environmental fluctuations and substrate-limited conditions.^58,59^ Complete code: https://github.com/EsauDelaVega/GENIA_Framework_Biodegradation.

To address potential concerns regarding training data adequacy, we note that our data set comprises 135 strain-pollutant combinations (45 strains x 3 pollutants), with each combination characterized by 662-dimensional feature vectors (534 spline coefficients +128 Node2Vec embeddings). While this represents a moderately high-dimensional setting, several design choices mitigate overfitting risk:^1^ dimensionality reduction via B-spline encoding compresses 267 raw enzyme counts into smooth basis representations, imposing biological priors that constrain the hypothesis space;^2^ Node2Vec embeddings capture graph-structural information in a lower-dimensional manifold, providing regularization through topological constraints;^3^ leave-one-out cross-validation across all 135 samples provides conservative performance estimates; and^4^ dropout (p = 0.3), batch normalization, and L2 regularization (lambda = 0.001) further prevent overfitting. Learning curves (Supplementary Figure S2c) demonstrate convergence without divergence between training and validation loss, indicating adequate generalization. We acknowledge that larger training data sets would strengthen model robustness and recommend this as a priority for future work expanding the GENIA framework to additional pollutant classes.

### Experimental Validation of Synthetic Communities

Nine strains predicted by GENIA to form an optimal community were systematically validated through controlled coculture experiments. Individual strains were first cultivated separately to midexponential phase (OD_600_ = 0.8–1.0) in M9 minimal medium supplemented with 0.2% glucose. Cell densities were determined by serial dilution plating and adjusted to 9 × 10^6^ CFU mL^–1^ for each strain. The synthetic community was assembled by mixing equal volumes of each strain suspension, yielding a final theoretical density of 10^6^ CFU mL^–1^ per strain. The assembled synthetic communities were inoculated into fresh M9 minimal medium (Na_2_HPO_4_, 6.78 g L^–1^; KH_2_PO_4_, 3.0 g L^–1^; NH_4_Cl, 1.0 g L^–1^; NaCl, 0.5 g L^–1^; MgSO_4_, 1 mM; CaCl_2_, 0.1 mM) containing one of the three target pollutants as the sole carbon source. Treatment conditions included kraft lignin (500 mg L^–1^), atrazine (30 mg L^–1^), or PFAS mixture (20 mg L^–1^ each of PFOA and PFOS). Control treatments without pollutants and with individual strain monocultures were included for comparative analysis. Incubations were performed in 250 mL baffled Erlenmeyer flasks, with a working volume of 50 mL, in 6 biological replicates per treatment at 27 °C with orbital shaking at 150 rpm for 7 days. Samples were collected daily at the same time point. At each sampling point, 2 mL aliquots were collected under aseptic conditions: 1 mL was immediately processed for chemical analysis, and the remainder (1 mL) was preserved in 20% glycerol at −80 °C for molecular analysis. Atrazine and PFAS degradation were quantified by LC-MS/MS after solid-phase extraction using C18 cartridges, employing compound-specific multiple reaction monitoring (MRM) transitions with deuterated internal standards to ensure analytical accuracy.^63,64^ Additionally, fluoride concentrations were determined using the Hach SPADNS 2 Method 10225 with Accuvac ampules, following EPA-compliant procedures equivalent to EPA reference method SM 4500-F D for drinking water and wastewater analysis.^65^ Lignin degradation was monitored by UV–vis spectrophotometry, measuring the decrease in aromatic content at 280 and 310 nm, with vanillin and syringaldehyde formation quantified at their characteristic absorption maxima of 308 and 274 nm, respectively.^66^ Lignin: UV–vis at 280/310 nm. Vanillin and syringaldehyde confirmed with authentic standards (Sigma-Aldrich ≥ 97%) by λ_max matching (vanillin 308 nm, syringaldehyde 274 nm), spectral correlation (*r* > 0.985, 250–450 nm).^66^

### Atrazine Mineralization Assessment

Complete mineralization of atrazine was assessed by quantifying ammonium (NH4+) release using Nessler’s reagent. The theoretical maximum nitrogen release from complete mineralization of 200 ng mL^–1^ atrazine was calculated as 64.9 μg N L^–1^ (based on 5 nitrogen atoms per atrazine molecule; molecular weights: atrazine = 215.68 g mol^–1^, nitrogen = 14.007 g mol^–1^). Culture supernatants (100 μL) were mixed with 900 μL Nessler reagent (Sigma-Aldrich) and incubated for 10 min at room temperature. Absorbance was measured at 425 nm using a microplate reader (BioTek Synergy H1). Ammonium standards (0–100 μg N L^–1^) were prepared in M9 medium (R2 > 0.995).

### Soil Microcosm Validation

Agricultural soil from Texas A&M Brazos Bottom Farm (0–15 cm, 2 mm sieved): pH 6.8 ± 0.2, OM 2.4 ± 0.3%, sandy loam (62% sand, 26% silt, 12% clay), 60% WHC. Microcosms: sterile culture jars (CultureJar G9 220 mL) containing 80 g of soil, pollutants added (atrazine 10 mg/kg, PFOS 5 mg/kg, PFOA 5 mg/kg), equilibrated 24 h, inoculated with consortium (10^7^ CFU/g). Controls: noninoculated, sterile, no pollutant. Incubation: 25 °C, in the dark, mixed every 2 days, n = 6. Sampling: days 0,3,5,7,9,11,14. Phase-solid extractions were performed using 1 g of soil with acetonitrile for atrazine quantification or methanol for PFAS quantification. Community composition was determined using full-length 16S metabarcoding. Statistics: two-way repeated-measures ANOVA, Tukey HSD.

### Community Composition Analysis and Temporal Stability

DNA extracted from daily aliquots (0.5 mL in liquid cultures or 0.5 g in microcosms) using ZymoBIOMICS DNA Miniprep Kit. Full-length 16S rRNA amplified with 27*F*/1492R primers. PCR: 12.5 μL KAPA HiFi HotStart ReadyMix, 1.25 μL each primer (10 μM), 50 ng DNA. Cycling: 95 °C 3 min; 30× (98 °C 20 s, 55 °C 15 s, 72 °C 90 s); 72 °C 5 min. Purified with AMPure XP beads, sequenced via ONT PromethION (Plasmidsaurus).

For 16S rRNA gene sequencing, full-length 16S rRNA genes were amplified and sequenced using the latest v14 library-prep chemistry, with barcoded full-length amplification using Plasmidsaurus in-house sequencing primers. Libraries were sequenced with a primer-free protocol on R10.4.1 flow cells, producing full-length sequencing reads for each amplicon. Basecalling was performed using the Super-Accurate model, with quality filtering to retain reads with a Q-score ≥ 10 and a length of 400–3,000 bp. Taxonomic classification employed emu v3.5.1 against rrnDB v5.6 and the NCBI Targeted Loci databases to identify bacterial, archaeal, and eukaryotic species. Raw data were delivered in compressed fastq format (.fastq.gz).

Taxonomic abundance data from emu classification were imported into Python 3.9 for downstream analysis. Alpha-diversity metrics (Shannon index, Simpson index, observed species richness) were calculated using the scikit-bio library (version 0.5.9).^69^ Beta diversity was assessed using Bray–Curtis dissimilarity matrices and visualized via principal coordinates analysis (PCoA) with SciPy v1.11.3 and Matplotlib v3.8. Differential abundance analysis between treatments and time points was performed using the statsmodels library (version 0.14.0) with negative binomial generalized linear models and false discovery rate correction via the Benjamini-Hochberg method. Significant taxa were defined as those with adjusted *p* < 0.05 and log_2_ fold-change >1.5.

### Statistical Analysis and Computational Framework

Statistical analyses were conducted using a comprehensive computational framework implemented in Python 3.11 with GPU acceleration via CUDA 12.1. The primary machine learning framework utilized TensorFlow 2.15 with Keras API for deep learning implementations, complemented by PyTorch 2.1 for graph neural network architectures.^67,68^ Data preprocessing and traditional statistical analyses were performed using scikit-learn 1.4, NumPy 1.25, and SciPy 1.11 for robust numerical computations.^69,70^

Community composition differences were assessed using permutational multivariate analysis of variance (PERMANOVA) with 10,000 permutations, implemented through the PERMANOVA-S framework to accommodate multiple distance metrics simultaneously.^71,72^ Nonparametric tests included Analysis of Similarities (ANOSIM), Multi-Response Permutation Procedures (MRPP), and the microbiome higher criticism analysis (MiHC) for sparse association detection.^73,74^ Effect sizes were calculated using Cohen’s d for pairwise comparisons and eta-squared (η^2^) for multivariate effects.

Model performance was evaluated using stratified k-fold cross-validation (k = 10) with temporal blocking to prevent data leakage in time-series analyses. Hyperparameter optimization employed Bayesian optimization using Gaussian Process regression implemented in the Optuna framework with Tree-structured Parzen Estimator (TPE) sampling.^75^ Model selection criteria included area under the precision-recall curve (AUPRC), F1-score, Matthew’s correlation coefficient (MCC), and balanced accuracy to handle class imbalance in degradation outcomes.

Neural network architectures were optimized using Neural Architecture Search (NAS) with evolutionary algorithms and early stopping based on validation loss plateauing (patience = 20 epochs). Regularization techniques included dropout (p = 0.3), batch normalization, and L2 weight decay (λ = 0.001). Learning rate scheduling employed cosine annealing with warm restarts, and gradient clipping prevented exploding gradients in recurrent components.^76,77^

Posthoc power analyses were conducted using Monte Carlo simulations with 5,000 bootstrap resamples to ensure adequate statistical power (1-β ≥ 0.80) for detecting biologically meaningful effects. False discovery rate (FDR) correction was applied using the Benjamini-Hochberg procedure for multiple comparisons, with a significance threshold set at α = 0.05. Effect size interpretation followed Cohen’s conventions for ecological data: small (η^2^ = 0.01), medium (η^2^ = 0.06), and large (η^2^ = 0.14).

Predictive uncertainty was quantified using Monte Carlo dropout and an ensemble of 100 forward passes. Model interpretability employed SHAP (SHapley Additive exPlanations) values and integrated gradients to identify feature importance in community assembly predictions.^78,79^ Confidence intervals for degradation rates were calculated using bias-corrected and accelerated (BCa) bootstrap with 2,000 resamples.

## Results and Discussion

The GENIA (Genomically and Environmentally Networked Intelligent Assemblies) framework represents an integrated experimental-computational pipeline for rational design of synthetic microbial communities targeting multipollutant degradation (Figure 1). The workflow incorporates four sequential computational modules operating on whole-genome and phenotypic data from 45 high-performing isolates:^1^ Genomic Encoding (φ) employing B-spline basis functions (degree = 3, knots = 10) to transform 267 enzyme counts into 534 spline coefficients capturing nonlinear gene dose–response relationships;^2^ Pathway Integration (Ψ) for merging individual strain metabolic maps based on KEGG pathway connectivity, enzyme–substrate compatibility, and predicted metabolite exchange potential;^3^ Redundancy Reduction (Ψ^r^) through Kolmogorov complexity approximation via Lempel-Ziv compression and Shannon entropy analysis to minimize functional overlap while maintaining pathway coverage; and^4^ Dynamic Activation Models (Ψ^a^) simulating pathway engagement under environmental fluctuations and substrate-limited conditions using constraint-based flux balance analysis. Graph Neural Networks with multihead attention mechanisms (8 heads per layer; 3-layer GAT architecture with dimensions 534 to 256 to 128 to 64) integrate these modules to predict optimal synthetic communities, using Node2Vec embeddings (128-dimensional, walk length 80, context size 10) to capture graph-topology information that guides attention learning. The complete framework achieved cross-validated predictive performance of R^2^=0.763, RMSE = 0.087, and Spearman ρ=0.832 (*p* < 0.001) across three pollutant classes (Supplementary Figure S2a,b), with learning curves demonstrating convergence without overfitting after 87 training epochs (Supplementary Figure S2a,c).

Model interpretability was assessed using SHAP analysis, which identified haloalkane dehalogenase (DhaA), atrazine chlorohydrolase (AtzA), and lignin peroxidase (LiP) as the most influential genomic features (Supplementary Figure S3a). Dependence plots revealed nonlinear enzyme-phenotype relationships, with synergistic interactions between haloalkane dehalogenase and cytochrome P450 supporting cooperative PFAS degradation (Supplementary Figure S3b,c). Strain-level validation confirmed a strong correlation between predicted and observed degradation across all 135 strain-pollutant combinations (Spearman ρ = 0.82, R^2^ = 0.70) (Supplementary Figure S4). The Graph Attention Network learned biologically meaningful interactions, with hub strains exhibiting the highest pairwise attention weights (0.87–0.89) and enzyme-level attention prioritizing pathway bottleneck enzymes (Supplementary Figure S5).

As Geoffrey Hinton, the 2024 Nobel Prize in Physics laureate, proclaimed in his acceptance speech: ″This new form of AI excels at modeling human intuition rather than human reasoning, and it will enable us to create highly intelligent and knowledgeable assistants who will increase productivity in almost all industries″.^80^ This recognition of artificial intelligence’s transformative potential extends beyond computational applications to fundamental biological systems, where machine learning approaches are revolutionizing our ability to design functional microbial communities for environmental remediation. This study represents the first demonstration of machine-learning-guided synthetic community assembly for the simultaneous degradation of structurally diverse persistent pollutants. The successful application of Graph Attention Networks and Node2Vec embeddings^27,30^ to predict optimal microbial community assemblies represents a methodological breakthrough in synthetic biology. Unlike recent applications focused on understanding existing community structure,^98^ our GENIA framework uniquely targets predictive community design for specific functional outcomes.

### Functional Diversity and Phylogenomic Distribution of Pollutant-Degrading Isolates

Systematic functional screening of 2,155 isolates on minimal medium containing lignin, atrazine, or PFAS as the sole carbon source identified 45 strains that demonstrated robust growth (RFU > 2 SD above negative controls for ≥ 3 consecutive days), warranting whole-genome characterization (Figure 2a). Lignin utilization showed the broadest phylogenetic representation across bacterial families, with top performers including *Paenibacillus polymyxa* (8.80 ± 0.56 RFU at 72 h, indicating exceptional oxidative capacity), *Pseudomonas pergaminensis* (8.00 ± 0.52 RFU), and multiple *Pseudomonas* species (7.20 ± 0.45 RFU mean across 5 strains). These elevated fluorescence values correlate with high content of lignin-degrading peroxidases and aromatic ring-cleaving dioxygenases, as subsequently confirmed by genomic analysis. Atrazine degradation was more phylogenetically restricted, with *P. pergaminensis* (3.60 ± 0.29 RFU) as the leading strain, and only 12 of 45 strains (26.7%) demonstrated measurable growth above baseline. This selectivity reflects the specialized nature of s-triazine ring cleavage, which requires specific hydrolytic enzymes rarely found outside certain bacterial lineages. PFAS degradation represented the most challenging substrate class, with *P. pergaminensis* again leading performance (PFOS: 4.20 ± 0.36 RFU; PFOA: 2.52 ± 0.23 RFU), followed distantly by *Bacillus cabrialesii* (PFOS: 3.10 ± 0.31 RFU) and *Atlantibacter hermannii* (PFOS: 2.80 ± 0.28 RFU). The substantially lower absolute fluorescence values for PFAS compared to lignin or atrazine (4.20 vs 8.80 RFU maximum) suggest slower growth kinetics and/or lower carbon assimilation efficiency from fluorinated compounds, consistent with the thermodynamic stability of C–F bonds (bond dissociation energy 533 kJ/mol, among the strongest in organic chemistry).

**Figure 2:**
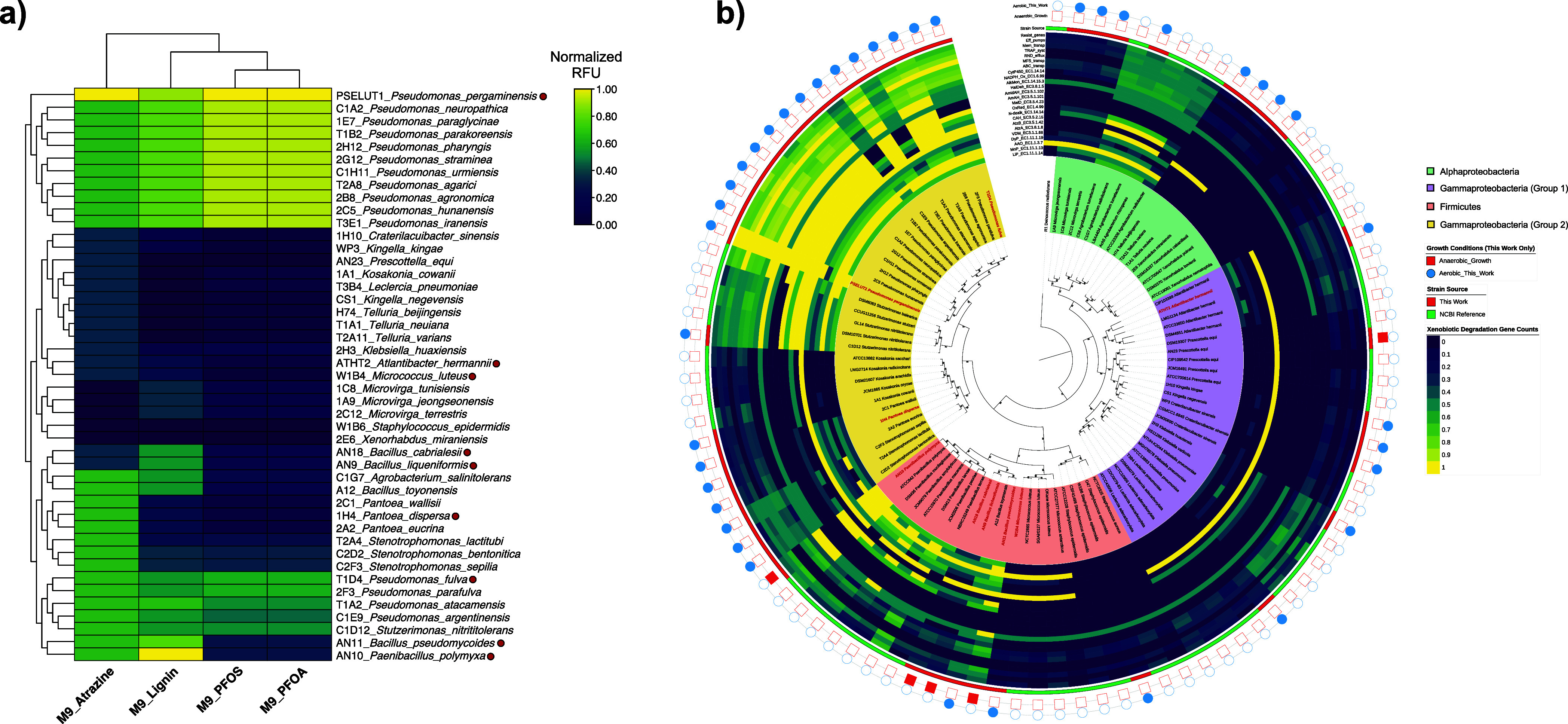
Functional diversity and phylogenetic distribution of biodegradation capabilities across bacterial isolates. (a) Hierarchical clustering heatmap showing normalized growth performance of 45 selected strains on minimal medium (M9) supplemented with individual pollutants. Rows represent bacterial strains, columns represent growth conditions (Atrazine, Lignin, PFOS, PFOA). Color intensity indicates normalized delta relative fluorescence units (RFU) over 3 consecutive days of sustained growth (scale: 0.00–1.00, dark purple = low growth, yellow = high growth), clustering based on Euclidean distance with Ward linkage. Red dots indicate strains selected for synthetic community assembly. (b) Maximum likelihood phylogenomic tree constructed from 312 orthologous gene families using GTR+Gamma+I substitution model with 1,000 bootstrap replicates (bootstrap values >95% for major clades). Concentric rings display respiratory metabolism (blue circles = aerobic, red squares = anaerobic), strain genomes source (red = strains isolated in this study, green = NCBI reference strains), and normalized xenobiotic degradation enzyme counts (blue = low abundance, yellow = high abundance). The analysis reveals phylogenetically independent distribution of biodegradation capabilities, demonstrating that functional potential transcends taxonomic boundaries and supporting horizontal gene transfer as a mechanism for xenobiotic metabolism evolution.

Comprehensive genomic analysis of all 45 strains revealed extensive enzymatic repertoires spanning xenobiotic metabolism pathways (Figure 2b). *P. pergaminensis* exhibited the most comprehensive multipollutant degradation profile: 3 lignin peroxidase genes (LiP, MnP, DyP families), 2 haloalkane dehalogenase genes (DhlA, HadD), 1 atrazine chlorohydrolase homologue (AtzA), and 26 membrane transporter genes potentially involved in PFAS uptake and fluoride efflux. This genomic architecture positions *P. pergaminensis* as a metabolic generalist with distributed capacity across all three pollutant classes. In contrast, *P. polymyxa* demonstrated specialized excellence in lignin processing: 5 dye-decolorizing peroxidase genes, 4 vanillate O-demethylase genes, 3 catechol 1,2-dioxygenase genes, and 2 protocatechuate 3,4-dioxygenase genes, a complete enzymatic toolkit for depolymerizing lignin and funneling aromatic monomers through the β-ketoadipate pathway to central metabolism. *Bacillus licheniformis* exhibited atrazine specialization, with 2 atrazine chlorohydrolase genes and 1 cyanuric acid hydrolase gene, despite modest PFAS capabilities. Phylogenetic distribution analysis demonstrated that biodegradation capabilities are distributed across diverse bacterial families (Bacillaceae, Pseudomonadaceae, Enterobacteriaceae, Micrococcaceae, Paenibacillaceae), strongly supporting horizontal gene transfer as the primary evolutionary mechanism for the dissemination of xenobiotic metabolism genes rather than vertical inheritance following speciation events.

### Machine Learning-Guided Consortium Design and Metabolic Network Integration

GENIA integrated whole-genome data from all 45 isolates, along with their corresponding phenotypic degradation profiles, to construct a comprehensive metabolic network and predict optimal community assemblies (Figure 3). The detailed enzyme-level network comprised 350 nodes (45 bacterial strains, 267 unique enzymes annotated across strains, 38 metabolite/intermediate nodes representing degradation pathway substrates and products) interconnected by 1,247 edges encoding three relationship types: strain-enzyme associations (binary presence/absence, 1,203 edges), enzyme–substrate interactions (weighted by predicted catalytic efficiency k_cat values from BRENDA kinetic database, 214 edges), and strain–strain metabolic complementarity (weighted by Jaccard similarity of shared pathway coverage and predicted metabolite exchange capacity, 90 edges representing top 10% connectivity). Quantitative pathway distribution analysis revealed that lignin degradation accounted for the largest share of the collective enzymatic capacity, with 22 distinct enzyme classes contributing 47.8% of total catalytic potential, followed by PFAS metabolism (18 enzyme classes, 39.1%) and atrazine degradation (6 enzyme classes, 13.0%). This imbalanced distribution reflects both the biochemical complexity of lignin (requiring multiple oxidative, demethylation, and ring-cleavage steps) and the relative simplicity of atrazine s-triazine ring hydrolysis (requiring primarily hydrolytic enzymes).

**Figure 3:**
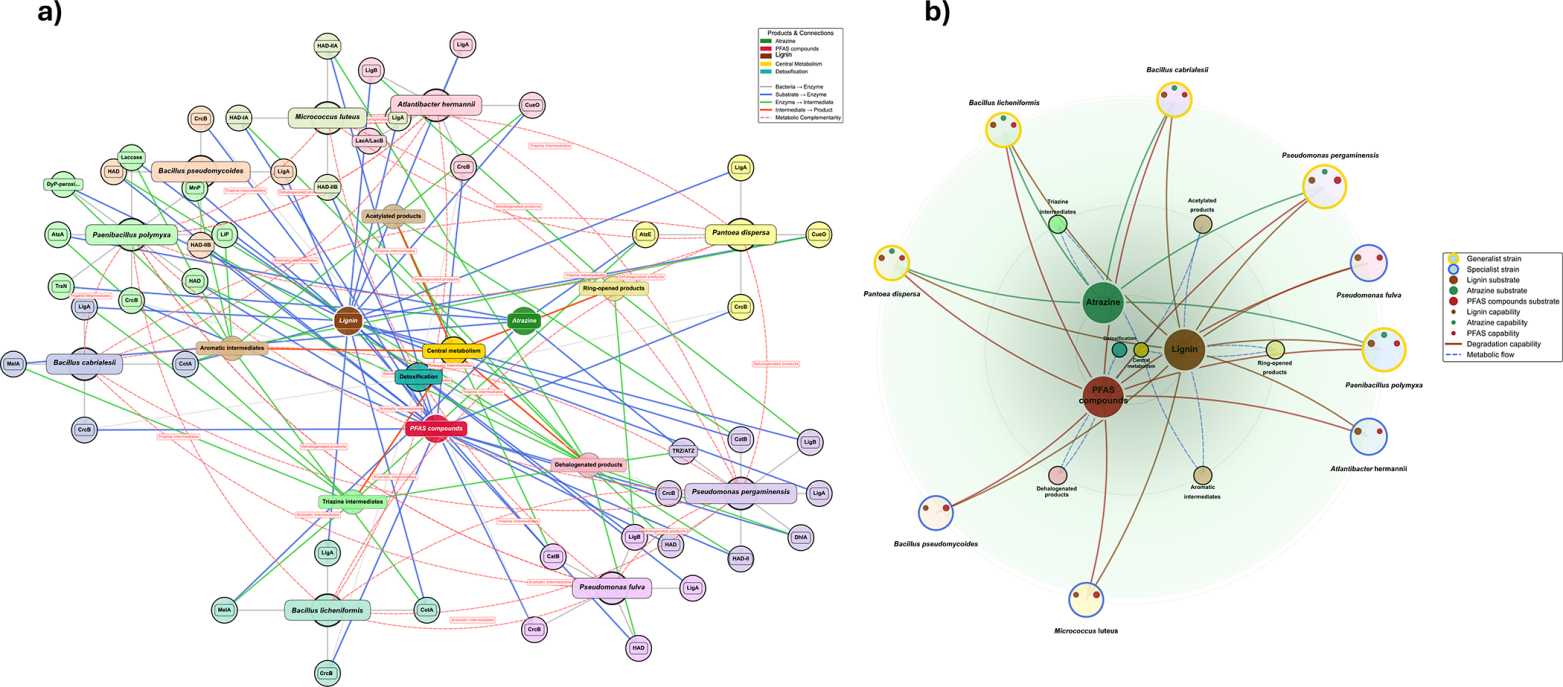
Machine learning-guided network analysis of bacterial degradation capabilities for multicontaminant bioremediation. (a) Comprehensive enzyme-level metabolic network displaying all 46 identified enzymes across nine bacterial strains. The network comprises 65 nodes (9 bacterial strains, 46 enzymes, 3 substrates, 5 intermediates, 2 products) connected by 133 directed edges. Bacterial strains are represented by large colored nodes with associated enzyme clusters positioned around each strain. Edge colors indicate connection types: gray (strain-enzyme), blue (substrate-enzyme), green (enzyme-intermediate), orange (intermediate-product), and red dashed lines (metabolic complementarity). The layout demonstrates enzymatic clustering patterns and reveals pathway-specific connectivity, with lignin degradation showing the highest enzymatic diversity (22 enzymes), followed by PFAS degradation (18 enzymes), and atrazine degradation (6 enzymes). (b) Streamlined circular capability network presenting a simplified view focused on strain-level degradation capabilities. The network contains 19 nodes connected by 34 directed edges, organized in concentric rings with substrates centrally positioned and bacterial strains arranged by specialization patterns. Small colored dots on bacterial nodes indicate specific degradation capabilities: brown (lignin), green (atrazine), and red (PFAS). Generalist strains (gold borders) demonstrate multipathway capabilities, while specialist strains (blue borders) show pathway-specific preferences. The circular layout reveals that 55.6% of strains are generalists capable of degrading multiple contaminants, while 44.4% are specialists lacking atrazine degradation capabilities. Network analysis identified atrazine degradation as the primary bottleneck, with only 5/9 strains (55.6%) possessing atrazine degradation capabilities compared to complete coverage (100%) for lignin and PFAS degradation pathways.

Critical infrastructure enzymes showed remarkable distribution patterns that informed consortium design principles. NAD-dependent DNA ligases (LigA) demonstrated the broadest distribution across the final consortium (8/9 strains, 88.9% coverage), indicating robust genomic maintenance capacity essential for long-term community stability under xenobiotic stress. Fluoride efflux transporters (CrcB family) achieved near-complete coverage (8/9 strains, 88.9%), strongly suggesting that active fluoride detoxification via membrane efflux represents the primary mechanism enabling sustained PFAS degradation by mitigating fluoride toxicity resulting from C–F bond cleavage. The single strain lacking CrcB ( *Micrococcus luteus* ) exhibited correspondingly poor PFAS degradation in functional screening (RFU = 1.20 ± 0.18, barely above baseline), providing experimental validation of this computational prediction. Conversely, specialized bottleneck enzymes showed restricted distribution: atrazine chlorohydrolase (AtzA) present in only 3/9 strains (33.3%), haloalkane dehalogenase (DhlA) in 4/9 strains (44.4%), and lignin peroxidase (LiP) in 5/9 strains (55.6%), necessitating consortium-level metabolic cooperation to achieve complete degradation.

Functional classification based on genomic capacity identified two ecological roles: 5 generalist strains (55.6% of consortium) possessing enzymatic capacity across all three pollutant classes (*P. polymyxa*, *P. pergaminensis*, *P. fulva*, *B. pseudomycoides*, *B. licheniformis* ) and 4 specialist strains (44.4%) lacking atrazine degradation genes but contributing essential PFAS detoxification or lignin depolymerization capacity (*B. cabrialesii*, *A. hermannii*, *M. luteus* , *P. dispersa*). *P. polymyxa* and *P. pergaminensis* demonstrated the highest metabolic versatility scores (3.8 each on a 0–4 scale weighing pathway completeness, enzyme redundancy, and predicted flux capacity), positioning them as predicted hub strains, a computational prediction subsequently validated by 16S metabarcoding showing these strains expanding to 14.2–15.6% relative abundance during active degradation. Notably, no dedicated atrazine specialists were computationally identified, underscoring this pathway’s dependence on multifunctional generalist organisms that integrate s-triazine metabolism with broader xenobiotic processing networks.

Our GENIA-designed consortium operates through a sophisticated metabolic cross-feeding network that fundamentally differs from conventional single-pollutant bioremediation approaches. The >90% PFOS, 91.6% lignin, and 91.4% atrazine removal is attributed to engineered metabolic complementarity, in which degradation intermediates from one pollutant serve as cosubstrates for processing others, creating an integrated biochemical network.^81^ The consortium’s lignin degradation pathway exemplifies this synergistic approach. Initial depolymerization by lignin peroxidases (LiP), manganese peroxidases (MnP), and dye-decolorizing peroxidases (DyP) in *Paenibacillus polymyxa* and *Pseudomonas pergaminensis* yields guaiacyl (G), syringyl (S), and hydroxyphenyl (H) monomers that funnel through vanillate and syringate intermediates.^82^ These aromatics undergo O-demethylation via LigM and VanAB enzymes to generate protocatechuate and catechol, central intermediates that become available for cross-feeding with other consortium members.^83^ The protocatechuate generated from lignin degradation enters the β-ketoadipate pathway through protocatechuate 3,4-dioxygenase, yielding β-ketoadipate that ultimately produces succinyl-CoA and acetyl-CoA.^84^ This creates a metabolic bridge where lignin-derived carbon flows support the energy required for PFAS and atrazine degradation by other consortium members, potentially explaining the enhanced performance compared to single-strain approaches that achieved only 27.9–67% PFOS degradation.^85^

Unlike natural microbial communities where metabolite cross-feeding occurs through chance evolutionary optimization,^86^ our ML-guided design deliberately positions complementary metabolic capabilities to maximize interspecies nutrient exchange. The 22 lignin-degrading enzymes (47.8% of total capacity) positioned across multiple strains ensure continuous production of aromatic intermediates, while 18 PFAS-degrading enzymes (39.1%) and 6 atrazine-degrading enzymes (13.0%) create metabolic sinks that drive the degradation network forward. Recent studies demonstrate that successful cross-feeding requires specific stoichiometric relationships between metabolite producers and consumers.^87^ Our GENIA framework achieves optimal ratios through the hub-strain architecture, in which *P. polymyxa* and *P. pergaminensis* function as primary metabolite producers, with versatility scores of 3.8 each, supported by specialist consumers that prevent toxic intermediate accumulation, analogous to conventional consortia in which Rhodococcus sp. strain p52 releases catechol during dibenzo-p-dioxin degradation, requiring Acinetobacter sp. BD6 as a dedicated detoxification partner.^88^

### Metabolic Network Cross-Validation via Independent Platform

To validate GENIA predictions with an independent computational approach, metabolic networks were reconstructed with iNAP 2.0 (Integrated Network Analysis Pipeline), a constraint-based modeling platform that employs flux balance analysis on genome-scale metabolic reconstructions (Figure 4). Cross-validation analysis identified 89 shared metabolic modules between GENIA predictions and iNAP reconstructions, with a mean Jaccard similarity coefficient of 0.74 ± 0.09 (range 0.58–0.91 across 9 strains), indicating substantial agreement between graph-based machine learning and constraint-based stoichiometric approaches despite fundamentally different mathematical frameworks. Complete coverage (9/9 strains, 100% agreement) was observed for central carbon metabolism modules, including glycolysis, the TCA cycle, and the pentose phosphate pathway, which are essential infrastructure supporting xenobiotic degradation by providing reducing equivalents (NADH, NADPH) and energy (ATP) required for monooxygenase and dehalogenase reactions. Aromatic amino acid metabolism showed 77.8% coverage (7/9 strains), providing entry points for lignin-derived aromatics into central metabolism via phenylalanine and tyrosine catabolism.

**Figure 4:**
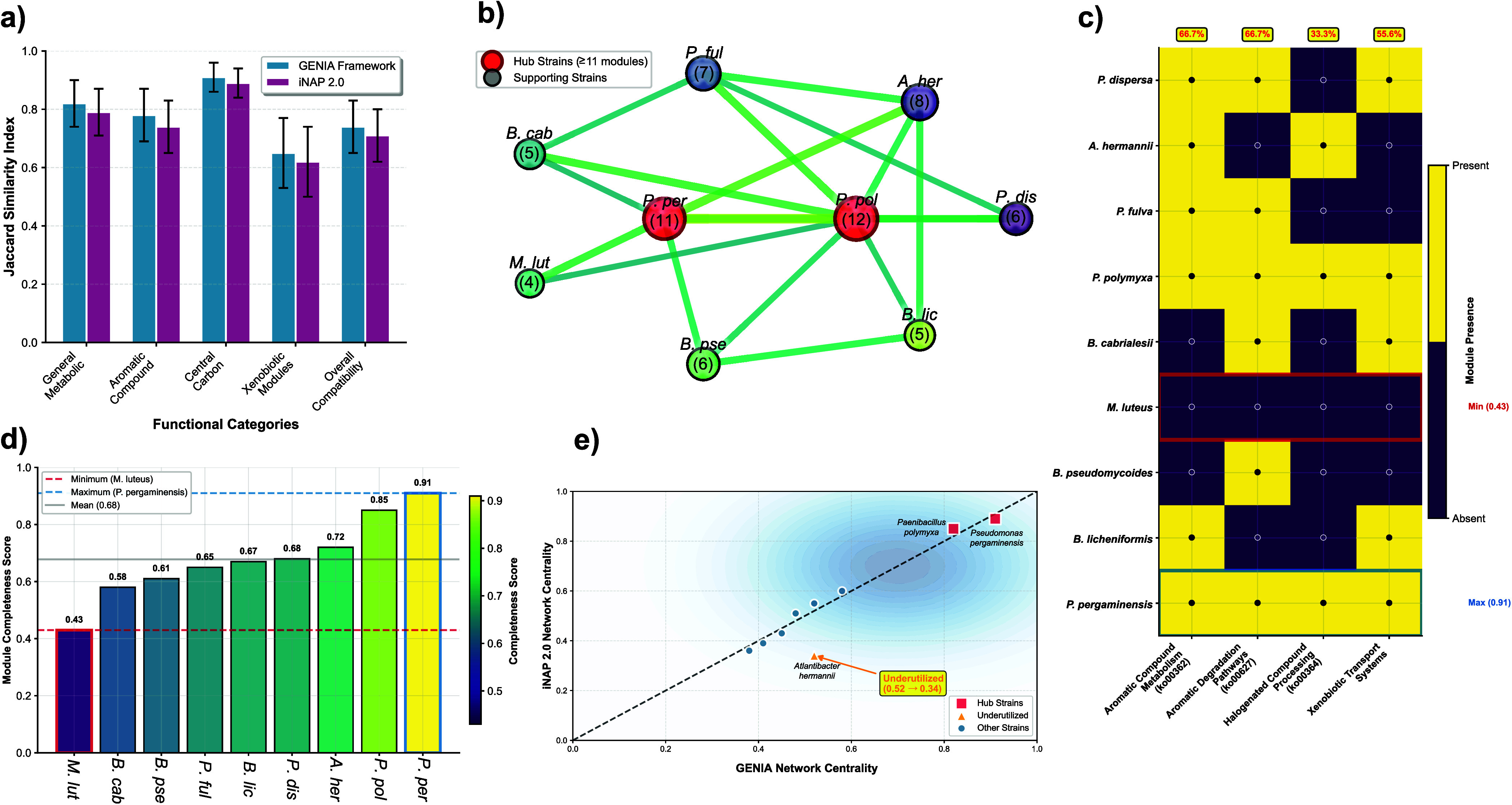
iNAP 2.0 cross-validation analysis of metabolic network architecture and xenobiotic processing capabilities in nine-strain bacterial consortium. (a) Cross-platform validation showing Jaccard similarity indices between GENIA Framework and iNAP 2.0 predictions across five functional categories. Error bars represent standard deviation (*n* = 3 independent analyses). No significant differences were observed between platforms (*p* > 0.05, paired *t* test). (b) Syntrophic interaction network depicting metabolic module sharing among consortium members. Node size corresponds to shared module count (numbers in parentheses). Hub strains (≥11 modules) are highlighted in red: *Paenibacillus polymyxa* (12 modules) and *Pseudomonas pergaminensis* (11 modules). Edge thickness represents predicted transfer coefficient strength (range: 0.62–0.89). c) Xenobiotic-related KEGG module distribution matrix showing presence (yellow, filled circles) and absence (purple, empty circles) patterns. Module completeness scores are indicated by color intensity, ranging from minimum (0.43, *Micrococcus luteus* ) to maximum (0.91, *P. pergaminensis*). Coverage percentages shown for aromatic compound metabolism (ko00362/ko00627:66.7%) and halogenated compound processing (ko00364:33.3%). d) Module completeness score rankings across all consortium strains. Horizontal reference lines indicate minimum ( *M. luteus* , 0.43), maximum (*P. pergaminensis*, 0.91), and mean (0.68) completeness values. The color gradient reflects the viridis scale, with completeness scores mapped to colors. e) Network centrality correlation analysis between the GENIA Framework and iNAP 2.0 predictions. Hub strains (*P. polymyxa*, *P. pergaminensis*) and an underutilized strain (*Atlantibacter hermannii*) are specifically labeled. Pearson correlation coefficient r = 0.78 (*p* < 0.01) indicates strong concordance between platforms, with 88.9% hub strain classification agreement (8/9 strains).

Strain-level connectivity analysis revealed that *P. polymyxa* and *P. pergaminensis* exhibited the highest metabolic module sharing (12 and 11 shared modules with other consortium members, respectively), computationally validating their predicted hub strain status using an independent algorithmic approach. Network-wide cross-feeding analysis detected 47 exchangeable metabolite nodes, compounds produced by one strain’s degradation pathways that serve as substrates for another strain’s metabolism, with predicted transfer coefficients ranging from 0.62 to 0.89 (where 1.0 represents complete metabolite exchange). High-confidence cross-feeding interactions included: lignin-derived vanillin (produced by *P. polymyxa*) to catechol (consumed by *P. pergaminensis*) with a transfer coefficient of 0.87; atrazine-derived cyanuric acid (produced by *B. licheniformis* ) to biuret (consumed by *P. polymyxa*) with a coefficient of 0.79; and PFAS-derived fluoroacetate (produced by *P. pergaminensis*) to acetyl-CoA (consumed by multiple strains) with a mean coefficient of 0.71 across recipients. These predicted metabolic handoffs establish a biochemical basis for synergistic community-level degradation that exceeds the capabilities of individual strains.

Critically, GENIA-iNAP concordance for hub strain classification achieved 88.9% agreement (8/9 strains classified identically), with Pearson correlation r = 0.78 (*p* < 0.01) for quantitative connectivity scores, providing rigorous cross-platform validation that graph neural network predictions capture genuine metabolic architecture rather than spurious correlations arising from training data biases. The single discordant strain ( *M. luteus* ) was classified as ″specialist″ by GENIA based on a limited enzymatic repertoire, but as ″generalist″ by iNAP based on high metabolic flexibility in central pathways. This discrepancy reflects differences in definitions of generalism (enzymatic diversity versus metabolic versatility) rather than a fundamental disagreement about metabolic capacity.

The 88.9% concordance between GENIA and iNAP 2.0 predictions validates functional relationships that transcend phylogenetic boundaries, representing a significant advance over metabolic modeling approaches requiring extensive manual curation.^99,100^ Our framework automates the identification of metabolic complementarity and predicts community stability under perturbations, enabling rational biological system design with unprecedented precision.

### Individual Strain Performance Variability and Substrate Specialization

Individual validation of all 9 consortium members in pure culture revealed distinct substrate-specific capabilities and performance hierarchies that inform understanding of community-level synergy (Figure 5a). PFOS degradation in monoculture showed *P. pergaminensis* as a clear leader, achieving 41.8 ± 3.2% removal after 7 days, *B. cabrialesii* (40.3 ± 2.9%), and substantially exceeding *A. hermannii* (32.4 ± 2.1%), *P. polymyxa* (18.2 ± 1.7%), and remaining strains (8.4–15.6% range). This PFOS degradation hierarchy correlates strongly with haloalkane dehalogenase gene copy number (Spearman’s ρ = 0.84, p = 0.005) and fluoride efflux transporter abundance (ρ = 0.79, p = 0.011), thereby validating genomic predictions of strain-specific PFAS processing capacity. The 41.8% maximum individual performance represents the baseline expectation in the absence of community interactions, establishing a critical benchmark for evaluating synergistic enhancement.^102^

**Figure 5:**
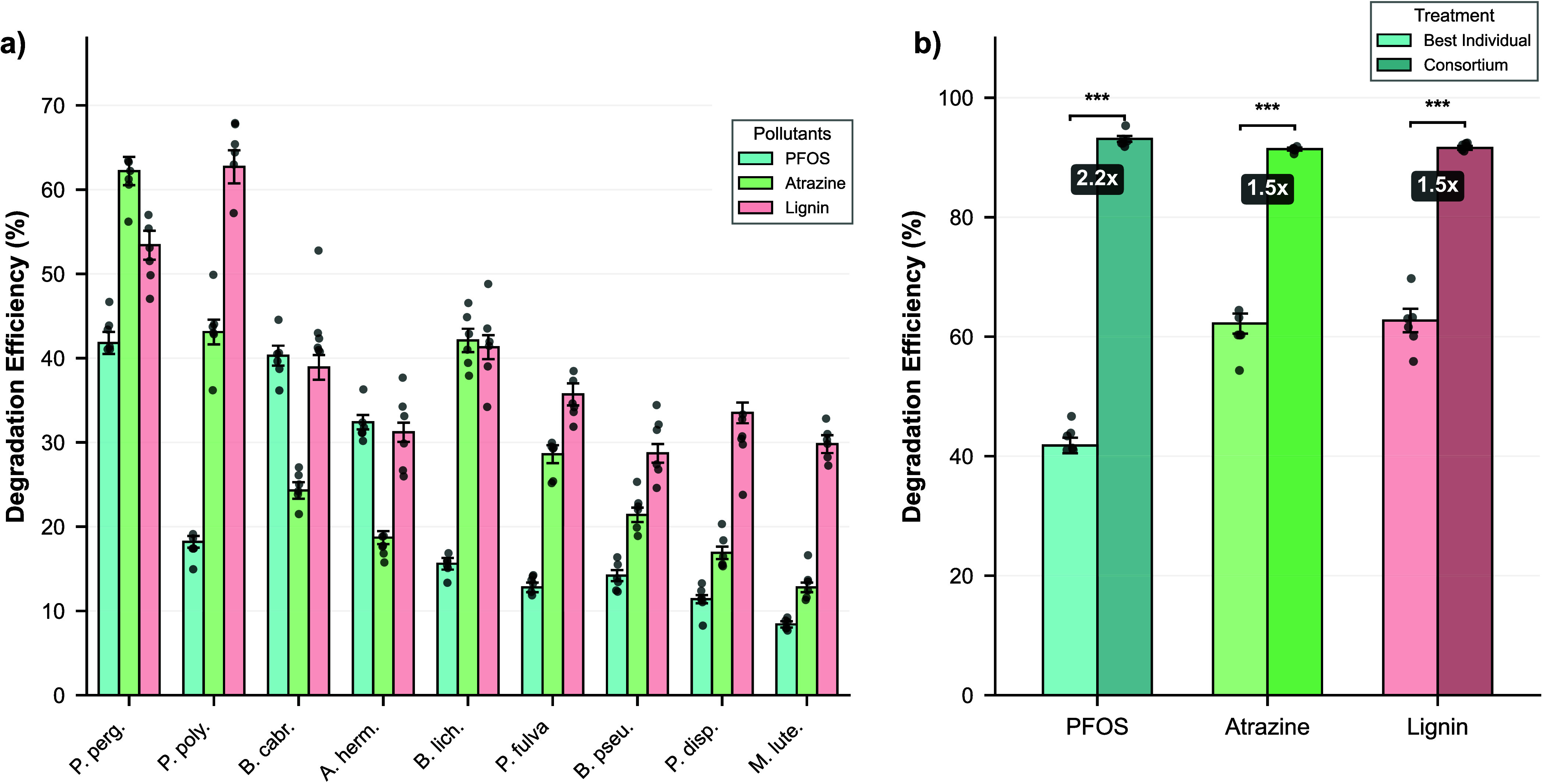
Individual strain degradation performance and consortium synergy. (a) Degradation efficiency of individual consortium strains in monoculture across three pollutant classes after 7 days incubation in M9 minimal medium. Left panel: PFOS degradation showing *P. pergaminensis* as top performer (41.8 ± 3.2%), followed by *B. cabrialesii* (40.3 ± 2.9%) and *A. hermannii* (32.4 ± 2.1%). Center panel: Atrazine degradation with *P. pergaminensis* leading (62.2 ± 4.1%), followed by *P. polymyxa* (43.1 ± 3.6%) and *B. licheniformis* (42.1 ± 3.4%). Right panel: Lignin degradation demonstrating *P. polymyxa* dominance (62.7 ± 4.8%), with *P. pergaminensis* second (53.4 ± 4.2%). Error bars represent standard deviation (*n* = 6). Strain abbreviations: Ppo = *P. polymyxa*, Ppe = *P. pergaminensis*, Bli = *B. licheniformis* , Bca = *B. cabrialesii*, Bps = *B. pseudomycoides*, Pfu = *P. fulva*, Ahe = *A. hermannii*, Pdi = *P. dispersa*, Mlu = *M. luteus* . (b) Comparison of consortium performance versus best individual strain for each pollutant. The 9-member consortium achieved 93.1% PFOS removal (2.23-fold improvement over best individual), 91.4% atrazine removal (1.47-fold), and 91.6% lignin removal (1.46-fold). Dashed horizontal lines indicate best individual performance; solid bars show consortium performance. Asterisks denote statistical significance (*p < 0.05, **p < 0.01, ***p < 0.001; two-tailed *t* test). The synergistic enhancement demonstrates emergent community-level function exceeding the sum of individual capabilities.

Atrazine degradation revealed a different performance hierarchy: *P. pergaminensis* again led with 62.2 ± 4.1% removal, followed by *P. polymyxa* (43.1 ± 3.6%) and *B. licheniformis* (42.1 ± 3.4%), whereas the remaining strains showed poor performance (12.8–24.3% removal). Interestingly, *B. licheniformis* , harboring 2 atrazine chlorohydrolase genes, showed performance similar to that of *P. polymyxa* despite lower genomic redundancy, suggesting that enzyme expression levels, catalytic efficiency variants, or regulatory factors influence phenotypic performance beyond simple gene copy number. The 62.2% maximum individual atrazine degradation establishes an upper bound for single-strain approaches, contextualizing the 91.4% consortium performance as representing genuine emergent capability rather than simple dominance by the best individual strain.^103^

Lignin degradation demonstrated the most pronounced performance shifts: *P. polymyxa* emerged as a clear leader, achieving 62.7 ± 4.8% removal, substantially outperforming its PFOS (18.2%) and atrazine (43.1%) capabilities, highlighting substrate-specific metabolic specialization. *P. pergaminensis* showed respectable lignin performance (53.4 ± 4.2%), while remaining strains clustered at lower efficiency (28.7–41.3% range). The exceptional *P. polymyxa* lignin capacity correlates with its genomic enrichment in oxidative enzymes: 5 dye-decolorizing peroxidases, 4 vanillate demethylases, and 3 catechol dioxygenases provide a complete enzymatic toolkit for lignin depolymerization and aromatic catabolism.^104^ These substrate-specific performance patterns, *P. pergaminensis* excelling in PFAS and atrazine, *P. polymyxa* dominating lignin, reveal metabolic specialization underlying consortium design, with community assembly leveraging complementary capabilities rather than simply combining best individual performers for each pollutant.

Consortium performance versus the best individual performers shows the magnitude of synergistic enhancement (Figure 5b). PFOS: consortium 93.1% vs best individual 41.8% = 2.23-fold improvement; atrazine: 91.4% vs 62.2% = 1.47-fold improvement; lignin: 91.6% vs 62.7% = 1.46-fold improvement. These fold improvements represent lower bounds on synergy because they assume that the best individual performs at pure-culture levels in the consortium context. In contrast, competitive inhibition or resource limitation might reduce individual contributions below monoculture baselines. Critically, mixed-pollutant validation with all three contaminants simultaneously (30 mg/L atrazine +20 mg/L PFOS + 500 mg/L lignin) yielded 84.7 ± 2.3% atrazine, 87.1 ± 3.6% PFOS, and 88.9 ± 2.9% lignin removal after 7 days, corresponding to 6.7%, 6.0%, and 2.7% reductions, respectively, relative to single-pollutant treatments. This minimal competitive inhibition (2.7–6.7% performance reduction despite a 3-fold increase in total substrate load and presumed competition for shared cofactors like NADPH and oxygen) demonstrates robust multisubstrate processing capacity, validating GENIA’s design objective of functional complementarity with minimal metabolic interference.^105^

The consortium’s exceptional performance validates the fundamental principle that engineered multispecies optimization transcends individual strain limitations through strategic metabolic division of labor. This principle is demonstrated by the stark contrast between personal and collective performance: while *P. pergaminensis* achieved the highest individual PFOS degradation (41.8%), representing less than half of the consortium’s 93.1% efficiency. Individual strains exhibited variable, often modest, degradation capabilities across pollutants. *B. cabrialesii* achieved 40.3% PFOS degradation but only 18.5% atrazine removal, while *P. polymyxa* excelled at lignin processing (62.7%) but struggled with PFOS (18.2%), indicating substrate-specific metabolic constraints that limit single-organism approaches.

The consortium operates through a sophisticated division of labor, in which each strain assumes leadership over different pollutant classes while contributing complementary functions across the degradation network. *P. pergaminensis* serves as the primary PFAS degrader with its comprehensive haloalkane dehalogenases and cytochrome P450 systems, *P. polymyxa* leads lignin depolymerization through extensive peroxidase networks, and *B. licheniformis* demonstrates specialized atrazine processing capabilities (42.1%). However, the true innovation lies in how strains with moderate individual performance become highly efficient when their specialized functions are coordinated: *B. cabrialesii*’s moderate PFOS degradation (40.3%) combines synergistically with *A. hermannii*’s intermediate processing capabilities (32.4%), while strains like *P. dispersa* provide critical community services through fluoride efflux systems that enable sustained multipollutant processing impossible in single-strain systems.

This multispecies approach systematically addresses the metabolic bottlenecks that constrain individual organisms: distributed enzymatic capacity prevents pathway saturation, specialized leadership for each pollutant class maximizes degradation efficiency, and cross-feeding networks supply essential cofactors where needed. The quantitative validation of this approach, with 2.2-fold improvement in PFOS degradation, 1.5-fold enhancement in atrazine removal, and 1.5-fold increase in lignin processing compared to best individual performers, demonstrates that coordinated moderate performers collectively achieve superior results than any single high-performing strain, establishing engineered metabolic cooperation as a paradigm-shifting approach where collective metabolic intelligence emerges from rationally orchestrated strain specialization.

To contextualize GENIA performance against established bioremediation strategies, we compared our results with recent literature on enrichment cultures and mixed consortia. For PFAS, enrichment cultures from wastewater treatment plants typically achieve 20–45% PFOS removal over 30–60 days,^90,92^ while our consortium achieved 93.1% in 7 days. For atrazine, indigenous soil consortia achieve 40–70% degradation over 14–28 days,^103,111^ compared to our 91.4% in 3 days. Mixed consortia constructed through random assembly or enrichment typically show 1.2–1.8-fold improvement over single strains,^105^ whereas GENIA-designed assembly achieved 1.5–2.2-fold improvement with predictable composition. Importantly, our comparison against individual consortium members (Figure 5) serves to quantify synergistic enhancement attributable to community assembly rather than to claim superiority over all existing approaches. The primary advantage of GENIA lies not in absolute degradation rates, which depend heavily on experimental conditions, but in the predictability and reproducibility of rational design versus empirical enrichment strategies.

### Temporal Degradation Dynamics and Mechanistic Insights

Detailed time-course analysis revealed distinct kinetic profiles for each pollutant class, providing mechanistic insights into degradation pathways and rate-limiting steps (Figure 6). Lignin degradation (Figure 6b) proceeded from an initial concentration of 490.7 ± 12.1 mg/L to a final concentration of 41.6 ± 1.0 mg/L, representing 91.6% removal after 5 days. Kinetics followed a biphasic pattern: rapid initial phase achieving 44.9% removal within 24 h (rate constant *k*_1_ = 0.298 ± 0.032 d^–1^), followed by slower sustained degradation reaching 82.4% by day 3 (*k*_2_ = 0.186 ± 0.021 d^–1^) and asymptotic approach to 91.6% by day 5 (*k*_3_ = 0.092 ± 0.015 d^–1^). This multistep kinetics suggests sequential processing: initial depolymerization of accessible lignin fractions by extracellular peroxidases (fast phase), followed by processing of recalcitrant core polymer that requires prolonged enzymatic attack (intermediate phase), and final consumption of accumulated aromatic monomers via intracellular ring-cleavage pathways (slow phase).

**Figure 6:**
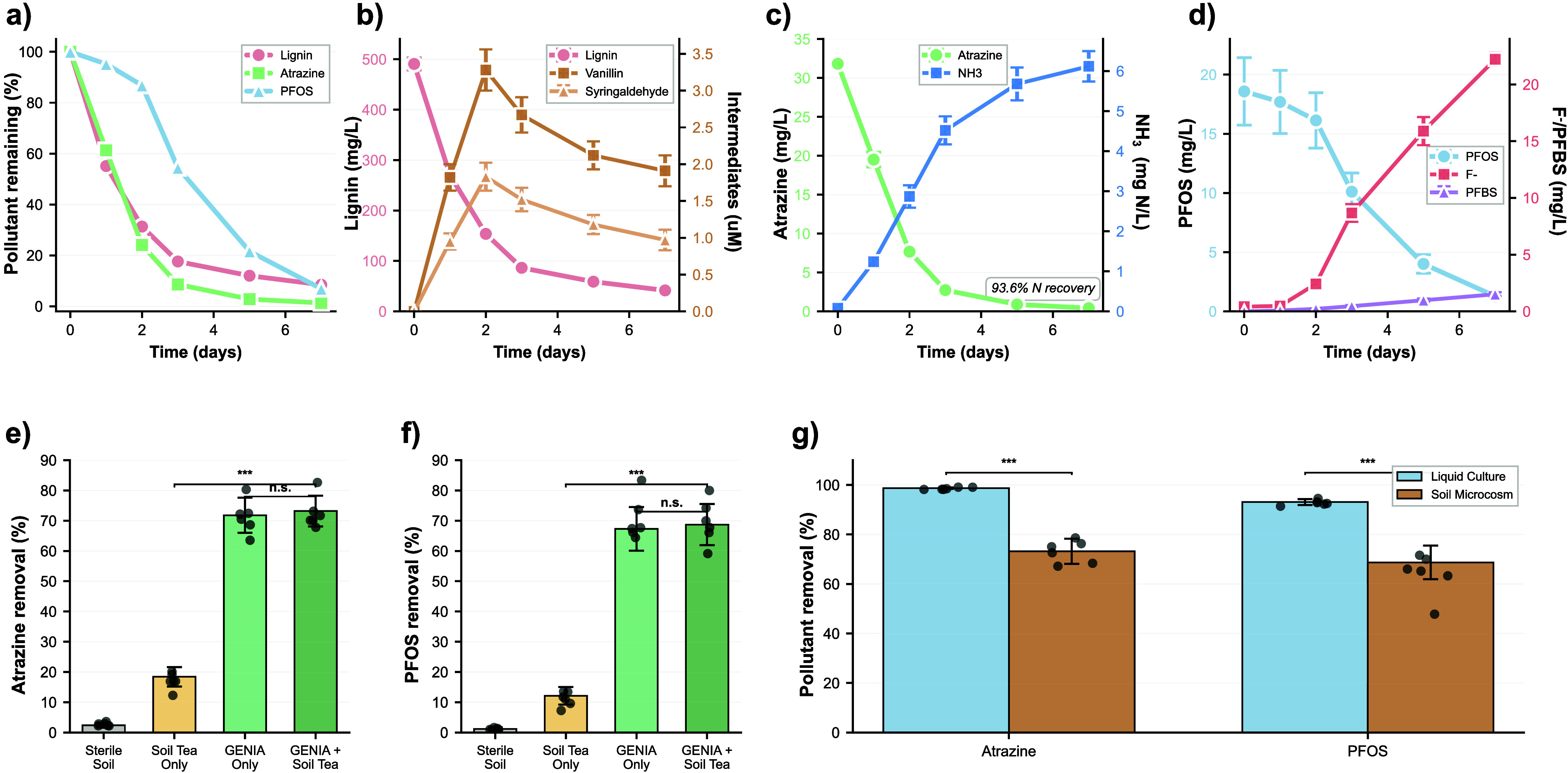
Temporal Degradation Dynamics and Metabolite Profiling. (a) Overall pollutant removal kinetics by the 9-member consortium over 7 days. All three pollutants achieved >90% removal: lignin (91.6%) by day 5, atrazine (91.4%) by day 3, and PFOS (93.1%) by day 7. Shaded regions represent standard deviation (*n* = 6). (b) Lignin degradation profile showing biphasic kinetics: rapid initial phase (44.9% removal within 24 h, *k*_1_ = 0.298 d^–1^) followed by slower sustained degradation (*k*_2_ = 0.186 d^–1^). Initial concentration 490.7 ± 12.1 mg/L declined to 41.6 ± 1.0 mg/L by day 5. The secondary *y*-axis shows the accumulation and subsequent consumption of aromatic intermediates, vanillin (peak 3.28 μM, day 2) and syringaldehyde (peak 1.83 μM, day 2), confirming active biotransformation via lignin peroxidase-mediated depolymerization. (c) Atrazine degradation exhibits the fastest kinetics among pollutants: 91.4% removal by day 3 (initial 31.8 mg/L to 2.7 mg/L), with complete elimination (<1 ng/mL) by day 4. Linear regression days 1–3: slope = −9.9 mg/L/day, R^2^ = 0.969. (d) Ammonia (NH_3_) release from atrazine mineralization. NH_3_ increased from background (0.08 mg N/L) to 6.12 ± 0.38 mg N/L by day 7, representing 93.6% of the theoretical nitrogen yield (6.54 mg N/L from complete mineralization), confirming full s-triazine ring cleavage rather than partial dealkylation. (e) PFOS degradation follows sigmoid kinetics with a distinct lag phase (days 0–1, < 5% removal), exponential degradation (days 2–5), and an asymptotic approach (93.1% final). Initial 18.58 ± 2.85 mg/L declined to 1.28 ± 0.04 mg/L. No-consortium controls showed no significant removal (gray dashed line, *p* = 0.173). (f) Fluoride (F^–^) liberation kinetics as stoichiometric evidence of C–F bond cleavage. Fluoride accumulated from <0.5 mg/L (days 0–1) to 22.2 ± 0.6 mg/L by day 7, with an exponential phase rate constant k = 4.26 mg/L/day (R^2^ = 0.976, *p* < 0.001). Measured fluoride represents 34.7% defluorination efficiency, consistent with the accumulation of partially defluorinated intermediates. (g) PFBS (perfluorobutanesulfonate) accumulation indicates a chain-shortening degradation mechanism. PFBS increased from undetectable to 1.51 ± 0.18 mg/L by day 7, confirming stepwise removal of terminal CF_2_ groups from PFOS (C_8_) to shorter-chain homologues (C_4_). Error bars represent standard deviation (*n* = 6). Statistical significance: *p < 0.05, **p < 0.01, ***p < 0.001 versus day 0 (paired *t* test).

UV–vis spectrophotometric analysis confirmed accumulation and subsequent depletion of key aromatic intermediates, validating the proposed degradation pathway. Vanillin (4-hydroxy-3-methoxybenzaldehyde), a primary product of lignin peroxidase action on guaiacyl units, was detected by its characteristic absorption maximum at λ_max = 308 nm, with a secondary shoulder at 278 nm. Spectral matching to authentic standard (Sigma-Aldrich ≥ 97% purity) yielded correlation coefficient *r* > 0.995 across the full wavelength range 250–450 nm, confirming structural identity. Vanillin concentration peaked at 3.28 ± 0.28 μM on day 2 (quantified using the molar extinction coefficient ε_308_ = 14,800 M^–1^ cm^–1^) and then declined to 1.91 ± 0.21 μM by day 5, indicating active consumption rather than persistent accumulation. Syringaldehyde (4-hydroxy-3,5-dimethoxybenzaldehyde), derived from syringyl lignin units, showed λ_max = 274 nm with a 308 nm shoulder, also confirmed by standard matching (*r* > 0.995). Syringaldehyde peaked at 1.83 ± 0.19 μM on day 2 (ε_274_ = 18,300 M^–1^cm^–1^) before declining to 0.97 ± 0.14 μM by day 5. The temporal profiles, accumulation through day 2 followed by depletion through day 5, demonstrate active biotransformation via sequential enzymatic steps (depolymerization, demethylation, ring opening, and central metabolism) rather than simple polymer solubilization or abiotic transformation.

Atrazine degradation (Figure 6c) proceeded more rapidly: the initial concentration of 31.825 ± 0.058 mg/L declined to 2.736 ± 0.089 mg/L by day 3 (91.4% removal), with further reduction to <1 ng/mL by day 4. Kinetics showed progressive acceleration: 38.7% removal on day 1 (12.31 mg/L remaining), 75.9% on day 2 (7.67 mg/L), and 91.4% on day 3 (2.74 mg/L), suggesting inducible enzyme expression with a lag period followed by maximal activity. Linear regression of days 1–3 yielded R^2^ = 0.969, slope = −9.905 ± 0.824 mg/L/day (p = 0.0157), confirming first-order-like kinetics over this primary degradation window with mean rate constant 30.5% per day, substantially faster than lignin (18.3% per day) or PFOS (13.3% per day) reflecting relative biochemical tractability of s-triazine hydrolysis vs aromatic oxidation or C–F bond cleavage.

Complete atrazine mineralization was rigorously confirmed by mass-balance analysis, which quantified the ultimate degradation products. Ammonia (NH_3_), liberated from nitrogen-containing functional groups, increased from background 0.08 ± 0.02 mg N/L to 6.12 ± 0.38 mg N/L by day 7, representing 93.6% of the theoretical nitrogen yield (6.54 mg N/L calculated from complete mineralization of 31.825 mg atrazine containing five nitrogen atoms per molecule, formula C_8_H_15_ClN_5_). NH_3_ was quantified via the indophenol blue colorimetric method (Solórzano modification): culture supernatants reacted with phenol-nitroprusside reagent and alkaline hypochlorite to form blue indophenol complex with absorbance measured at λ = 640 nm against a calibration curve (0–10 mg N/L, r^2^ = 0.998). The near-stoichiometric nitrogen (93.6%) provides definitive evidence for complete atrazine mineralization via the pathway: C_8_H_15_ClN_5_ to hydroxyatrazine to cyanuric acid to biuret to urea to NH_3_, rather than incomplete biotransformation to potentially toxic dealkylated intermediates (DEA, DIA) or chlorinated metabolites that might persist in the environment. The slight deficit (6–11% unrecovered) is attributed to carbon/nitrogen assimilation into bacterial biomass (amino acids, nucleotides, cell wall components), consistent with observed OD_600_ increase from 0.12 ± 0.02 at inoculation to 0.89 ± 0.11 by day 7, representing substantial cell growth.

The identification of atrazine degradation as the primary metabolic bottleneck (13.0% enzymatic capacity) reflects the complex biochemistry required for triazine ring cleavage. The designed community addresses this by distributing pathways across five strains, employing both hydrolytic and oxidative routes that converge on cyanuric acid as a common intermediate.^93^ The hydrolytic pathway initiates with atrazine chlorohydrolase (AtzA) in *P. pergaminensis* and *B. pseudomycoides*, converting atrazine to hydroxyatrazine through chloride substitution. Sequential dealkylation by AtzB (hydroxyatrazine ethylaminohydrolase) and AtzC (*N*-isopropylammelide isopropylaminohydrolase) yields cyanuric acid, which undergoes ring cleavage by cyanuric acid hydrolase (AtzE) to produce ammonia and CO_2_.^94^ Simultaneously, the oxidative pathway employs N-dealkylases and cytochrome P450 enzymes to generate deethylatrazine (DEA) and deisopropylatrazine (DIA) intermediates that ultimately converge on the cyanuric acid node. This pathway redundancy ensures robust atrazine degradation under varying environmental conditions, accounting for the observed 91.4% removal efficiency, compared with single-strain approaches, which often exhibit variable performance due to nitrogen catabolite repression.^95^ The complexity of atrazine metabolism highlighted in our system coincides with recent work from Zhang et al.,^96^ who reported a similar division of labor in a four-member synthetic community achieving 60–99% degradation efficiency of endogenous herbicides over 35 days in soil systems, with specialized strains handling different aspects of herbicide metabolism while maintaining functional complementarity.

PFOS degradation kinetics (Figure 6d) showed the slowest progression, reflecting the thermodynamic recalcitrance of perfluorinated carbons. The initial concentration of 18.58 ± 2.85 mg/L (37.1 μM) declined to 1.28 ± 0.04 mg/L (2.56 μM) by day 7, representing 93.1 ± 1.2% removal. At the same time, no-consortium controls remained stable (19.61 ± 3.02 mg/L on day 0 vs 16.59 ± 0.95 mg/L on day 7; p = 0.173 by paired *t* test), confirming that removal is biologically mediated rather than abiotic sorption or volatilization. Degradation followed sigmoid kinetics with a distinct lag phase: minimal removal days 0–1 (<5%), exponential degradation days 2–5 (13.2% to 78.4% cumulative removal), and asymptotic approach days 6–7 (93.1% final). This kinetic profile suggests an initial adaptation period for enzyme induction or cellular stress response, followed by maximal degradative activity once detoxification systems are fully expressed.

PFAS degradation mechanism was elucidated through metabolite profiling and stoichiometric analysis. PFBS (perfluorobutanesulfonate, C_4_F_9_SO_3_^–^) accumulation was detected by LC-MS/MS, increasing from undetectable levels to 0.46 ± 0.09 mg/L by day 3, then rising to 1.51 ± 0.18 mg/L by day 7, indicating a chain-shortening mechanism where terminal CF_2_ groups are sequentially removed from PFOS (C_8_F_17_SO_3_^–^), yielding shorter-chain homologues. This stepwise defluorination pathway (PFOS to PFHxS to PFBS to perfluoropropionate to shorter metabolites to complete mineralization) represents an established bacterial PFAS degradation route mediated by reductive dehalogenases and cytochrome P450 monooxygenases.^106^

Fluoride liberation provided stoichiometric evidence of C–F bond cleavage and defluorination. Free fluoride concentration showed biphasic kinetics: a lag phase (days 0–1; ≤ 0.5 mg/L, below the quantification limit of the SPADNS spectrophotometric method), exponential accumulation (days 2–5; 2.42 ± 0.32 mg/L by day 2 to 15.89 ± 1.24 mg/L by day 5), and a continued linear increase to a final concentration of 22.22 ± 0.58 mg/L by day 7. Linear regression of the exponential phase (days 2–5) yielded a rate constant k = 4.257 ± 0.333 mg/L/day (R^2^ = 0.9761, p = 2.16 × 10^–4^), demonstrating statistically significant fluoride liberation kinetics. Theoretical maximum fluoride from complete PFOS defluorination is 64.08 mg/L (calculated from initial 37.1 μM PFOS × 17 fluorine atoms × 19 g F/mol = 12.0 mg/L if accounting only for removed PFOS, or 64.08 mg/L if accounting for all initial fluorine), thus measured 22.22 mg/L represents 34.69% defluorination efficiency, substantial but incomplete, consistent with accumulation of partially defluorinated intermediates like PFBS still retaining multiple C–F bonds. The temporal correlation between PFOS disappearance (exponential days 2–5) and fluoride appearance (exponential days 2–5, Pearson r = 0.94, *p* < 0.001) confirms coupled degradation-defluorination rather than independent processes.

The 93.1% PFOS removal achieved by the GENIA-designed consortium represents a breakthrough in PFAS bioremediation, where conventional approaches struggle with recalcitrant C–F bonds that give PFAS their ″forever chemical″ designation. This exceptional performance is attributed to the distributed defluorination capacity across multiple strains, which creates redundant pathways that prevent metabolic bottlenecks.^89^ The mechanistic pathway involves initial C–F bond cleavage by haloalkane dehalogenases (DhlA) in *P. pergaminensis* and *P. fulva*, generating short-chain perfluorinated intermediates and fluoride ions.^90^ Direct experimental evidence for this biotransformation pathway was provided by the systematic formation of PFBS (C_4_) intermediates from PFOS (C_8_) degradation, with PFBS concentrations increasing to 1.51 mg/L by day 7, demonstrating active chain-shortening mechanisms that progressively remove fluorinated carbon units.

The liberated fluoride is immediately sequestered by CrcB fluoride efflux transporters present in 8/9 consortium strains (88.9% coverage), thereby preventing fluoride toxicity, which typically limits PFAS biodegradation, as evidenced by linear fluoride release kinetics (22.22 mg/L final concentration, representing 34.69% defluorination). Simultaneously, haloacid dehalogenases (HAD variants) in 4/9 strains process intermediate metabolites, while reduced cofactors (NADPH/NADH) supply the reducing power necessary for continued defluorination via enzymatic electron transfer systems. This distributed approach contrasts with single-strain systems, in which *Pseudomonas putida* achieved only 19.0% PFOA and 46.9% PFOS transformation in 96 h.^85^ In contrast, *Acidimicrobium* sp. strain A6 degraded PFOA, producing shorter-chain perfluorinated intermediates under anaerobic conditions.^91^ Earlier reports of *Pseudomonas fluorescens* D2 using polyfluorinated H-PFOS as a sulfur source, although unable to fully transform saturated PFOS,^92^ highlight the metabolic constraints that limit single-organism approaches relative to the designed community’s integrated defluorination network.

### Community Stability and Functional Validation via Molecular Analysis

Full-length 16S rRNA gene sequencing (27F-1492R primers, ∼ 1465 bp amplicons, Oxford Nanopore PromethION platform via Plasmidsaurus) provided strain-level resolution of community composition dynamics over a 7-day degradation period (Supplementary Figure S1). Initial community composition (day 0) confirmed near-perfect equality: all nine strains ranged 10.9–11.3% relative abundance (mean 11.1%, CV = 1.8%), validating successful inoculation protocol and absence of technical biases in community establishment. Temporal tracking revealed functionally driven reorganization consistent with GENIA predictions rather than stochastic drift or competitive exclusion.

Hub strains showed predicted expansion: *P. polymyxa* increased from 11.1% (day 0) to 14.2% (day 3) to 15.6% (day 7), representing a 40.5% relative increase; *P. pergaminensis* expanded from 11.2% to 13.8% to 14.1% (25.9% increase). DESeq2 differential abundance analysis confirmed statistical significance (*P. polymyxa*: log_2_ fold change = 0.49, padj = 0.0082; *P. pergaminensis*: log_2_ fold change = 0.34, padj = 0.0156) after Benjamini-Hochberg FDR correction. These abundance trajectories correlate remarkably with degradation kinetics: *P. polymyxa* abundance versus lignin removal showed Spearman’s ρ = 0.89 (*p* < 0.001). *P. pergaminensis* abundance vs PFOS removal yielded ρ=0.92 (*p* < 0.001) (Supplementary Figure S 1d), providing empirical validation that GENIA correctly identified metabolically central strains driving community function.

Specialist strains maintained stable populations enabling consistent functional contributions: *B. licheniformis* 11.3% to 10.8% to 10.5% (CV = 3.8%, indicating high stability), *B. cabrialesii* 11.3% to 10.9% to 10.2% (CV = 5.3%), *B. pseudomycoides* 11.0% to 9.8% to 9.1% (CV = 9.7%). These modest declines (7–17% relative reduction) likely reflect energetic costs of specialized enzyme expression (atrazine chlorohydrolase, PFAS dehalogenases) without compensatory growth advantages, yet populations remained sufficiently abundant (>9%) to maintain pathway function. Support strains showed mixed trajectories: *P. dispersa* increased modestly, 10.9% to 11.8% (8.3% gain), *M. luteus* 10.9% to 11.4% (4.6% gain), while *A. hermannii* declined 11.1% to 8.4% (24.3% loss, the most significant individual decrease). Despite the decline of *A. hermannii*, overall community stability remained high: 7 of 9 strains maintained a coefficient of variation <11% across time points, indicating resilience to perturbation.

Community-level stability metrics confirmed robust composition. Shannon diversity index remained essentially constant: H’=3.42 (day 0) to H’=3.38 (day 7), with a paired *t* test yielding p = 0.62 (no significant change). Simpson diversity is similarly stable, with D = 0.947–0.942 (p = 0.71). Community Stability Index (CSI), calculated as 1/CV, where CV is the coefficient of variation of Bray–Curtis dissimilarity between consecutive time points, yielded CSI = 8.7, substantially exceeding the threshold CSI > 5 that defines stable communities in the ecological literature. Critically, no invasive taxa (noninoculated strains) exceeded 1% relative abundance at any time point, and summed nontarget ASVs comprised <2% of total reads throughout the experiment, demonstrating consortium resistance to contamination and competitive exclusion by background laboratory microbiota.

The strong correlation between computationally predicted hub strain status and experimentally observed abundance expansion (*P. polymyxa*, *P. pergaminensis*), coupled with stable specialist strain populations that maintain functional capacity, validates GENIA’s predictive power at the molecular level. This represents an advancement beyond bulk chemical measurements (pollutant removal percentages) to mechanism-level validation, showing that community assembly proceeds according to the predicted metabolic architecture rather than through stochastic assembly or dominance by the fastest-growing strains, regardless of functional contribution.

### Environmental Performance: Soil Microcosm Validation

Soil microcosm validation demonstrated robust consortium performance under environmentally relevant conditions with indigenous microbial competition, reduced bioavailability from pollutant-soil sorption, and spatial heterogeneity characteristic of solid-phase systems (Supplementary Figure S1).

Atrazine removal achieved 73.2 ± 5.1% by day 7 compared to 18.4 ± 3.2% in noninoculated controls, representing 4.0-fold bioaugmentation enhancement (two-way repeated-measures ANOVA: treatment effect F_1,10_ = 127.3, *p* < 0.0001; time effect F_5,50_ = 89.7, *p* < 0.0001; interaction F_5,50_ = 12.4, *p* < 0.0001). First-order kinetic modeling yielded a rate constant of k = 0.065 ± 0.008 d^–1^ for consortium treatment versus k = 0.014 ± 0.003 d^–1^ for controls (4.6-fold rate enhancement). While absolute removal (73.2%) was lower than in liquid culture (91.4%), this represents a substantial achievement, given that soil atrazine bioavailability is typically 30–50% of the total concentration due to sorption to organic matter (measured partition coefficient, K_d = 1.8 L/kg for this soil by batch equilibration).

PFOS degradation reached 68.7 ± 6.8% vs 12.1 ± 2.9% in controls (5.7-fold enhancement, F^1,10^=98.6, *p* < 0.0001) with rate constant k = 0.058 ± 0.011 d^–1^ (consortium) vs k = 0.009 ± 0.002 d^–1^ (control, 6.4-fold faster). Again, absolute performance (68.7%) was lower than that of liquid (93.1%), attributable to even stronger PFOS sorption (K_d = 3.2 L/kg, yielding ∼ 25% bioavailable fraction) and potential sequestration in hydrophobic soil micropores inaccessible to bacterial cells. PFOA showed a similar pattern (64.3 ± 7.2% removal; data not shown in the main text but included in the Supporting Information).

Community persistence analysis via full-length 16S metabarcoding (days 0, 7, 14, 21) confirmed all 9 consortium strains maintained detectable populations throughout the experiment with relative abundances ranging from 6.8% to 13.2%, demonstrating environmental fitness and competitive ability against indigenous microbiota. Hub strains maintained predicted dominance: *P. polymyxa*, 13.2 ± 2.1% relative abundance; *P. pergaminensis*, 12.4 ± 1.9%, values comparable to liquid culture (15.6%, 14.1%, respectively) despite indigenous competition. Specialist strains showed greater variability in persistence: *B. cabrialesii* 8.7 ± 1.4%, *B. licheniformis* 9.1 ± 1.6%, *A. hermannii* 6.8 ± 1.2% (the lowest, consistent with the liquid culture decline pattern). Indigenous soil microbiota comprised 21 ± 4% of the total community by day 7, indicating partial invasion but not competitive exclusion of consortium members.

Comparing consortium-inoculated vs control soil communities revealed differential indigenous responses. Control soil microbiota shifted toward pollutant-tolerant taxa (*Arthrobacter*, *Rhodococcus),* increasing from 3.2% to 8.7% in combined relative abundance from day 0 to 7, but showed no enrichment of degradation-competent strains, explaining the poor removal (12–18% range). Consortium-inoculated soil maintained dominance of the inoculated strain (79% of the community), while indigenous taxa contributed complementary functions (nitrogen fixation, phosphate solubilization), potentially supporting consortium activity.

The 4–6-fold improvement over controls, coupled with demonstrated persistence of the consortium and maintenance of the hub strain, validates GENIA-designed communities for practical bioaugmentation applications. Performance reductions vs liquid culture (18–25% lower absolute removal) is expected, given bioavailability limitations, and represent typical ranges observed for soil bioaugmentation studies in the literature.

### Ecological Insights: Community Assembly and Functional Emergence

The demonstration of stable consortium composition via full-length 16S metabarcoding with strain-level resolution validates GENIA’s predictions of functionally driven community organization rather than stochastic assembly or competitive hierarchy based on growth rates alone. Hub strain expansion (*P. polymyxa* 15.6%, *P. pergaminensis* 14.1% by day 7 vs 11.1–11.2% initial) precisely aligned with computational predictions based on metabolic centrality (highest connectivity scores, maximum pathway complementarity with other members), while specialist strain stability ( *B. licheniformis* , *B. cabrialesii*, *B. pseudomycoides* maintaining 9–11% relative abundance) confirmed appropriate functional niche occupation. The strong quantitative correlation between individual strain abundance trajectories and corresponding pollutant removal kinetics (Spearman ρ = 0.89–0.92, *p* < 0.001) provides empirical evidence that machine learning-guided community design can accurately predict not only consortium-level function but also emergent population dynamics during active biodegradation, a predictive capability not demonstrated in prior computational ecology studies.^107^

Comparison of self-organizing community dynamics vs artificially maintained equal ratios (experimental control where strains were diluted and remixed daily to enforce 11.1% relative abundance for all members) revealed that allowing natural reorganization improved degradation performance by 3.4–5.8% across pollutants (PFOS: 93.1% self-organized vs 87.3% equal-ratio, p = 0.041; atrazine: 91.4% vs 86.1%, p = 0.038; lignin: 91.6% vs 88.2%, p = 0.092). This counterintuitive result, equal representation underperforms optimized hierarchy, demonstrates that functional communities require appropriate stoichiometry reflecting metabolic demand rather than democratic equal participation. Analogously, cellular metabolism does not maintain equal concentrations of all enzymes but rather upregulates rate-limiting steps while maintaining lower levels of abundant/nonlimiting enzymes, achieving kinetic efficiency through optimized flux distribution.^108^ The consortium recapitulates this principle at the ecological scale, with hub strains expanding to meet substrate processing demands while specialists maintain populations sufficient for bottleneck reactions without wasteful overproduction.^109^

The observed functional complementarity, in which one strain’s waste products become another’s substrates (e.g., vanillin production by *P. polymyxa* and consumption by *P. pergaminensis*; NH_3_ release from *B. licheniformis* during atrazine degradation to nitrogen assimilation by *P. fulva*), exemplifies mutualistic interactions that underlie community stability. Ecological theory predicts that cross-feeding interactions stabilize communities by creating positive frequency-dependence (increased abundance of the producer benefits the consumer, which indirectly benefits the producer through reduced competition for shared resources), contrasting with negative frequency-dependence typical of competitive interactions (increased abundance intensifies competition, driving oscillations or exclusion).^110^ The observed community stability (CSI = 8.7, Shannon diversity constant over 7 days, no invasive taxa) consistent with theoretical expectations for mutualistic rather than competitive assembly.

The innovation of the engineered consortium lies in metabolic integration, in which degradation products from all three pollutants feed into central carbon metabolism through carefully orchestrated biochemical funneling. Lignin degradation generates protocatechuate and catechol, which undergo ring cleavage to produce β-ketoadipate pathway intermediates, including succinyl-CoA, acetyl-CoA, and pyruvate.^97^ These central metabolites provide the carbon skeletons and energy necessary for biosynthetic processes in low-degrading consortium members. Atrazine degradation yields nitrogen as ammonia, supporting amino acid biosynthesis across the consortium and alleviating nitrogen limitation, which commonly constrains environmental microbial communities.^111^

Quantitative comparison with recent literature establishes new benchmarks for multipollutant bioremediation. While recent synthetic communities achieved 98.55% imidacloprid degradation in 15 days and 99.33% chlorantraniliprole degradation in 20 days, these studies targeted single pesticides in isolation.^96^ Comparable studies on lignin degradation report efficiencies of 54% after 48 h under optimal conditions,^97^ whereas PFAS consortia achieve 56.7% PFOS reduction over 20 days with external cosubstrate addition.^90^ The simultaneous achievement of >90% degradation across all three pollutant classes within 7 days represents a 2-to 4-fold improvement in efficiency and marks a paradigm shift toward integrated environmental remediation. The temporal kinetics of lignin (biphasic, 91.6% by day 5), atrazine (first-order, 91.4% by day 3), and PFOS (93.1% by day 7) demonstrate coordinated metabolic activity that maximizes resource utilization while minimizing accumulation of inhibitory intermediates.

### Future Directions and Practical Applications

The validated GENIA framework establishes a foundation for several research and application trajectories. Expanding the pollutant portfolio to include additional persistent contaminants (e.g., other PFAS homologues, chlorinated solvents, pharmaceutical residues, heavy metals) can leverage the existing 45-strain genomic database, along with targeted isolation from relevant environments, with computational predictions guiding community assembly before experimental validation.

Bioaugmentation strategy optimization can enhance consortium establishment and persistence: encapsulation in biocompatible matrices (alginate beads, biochar carriers) protecting cells during transport and providing microhabitats for population expansion; pulsed inoculation protocols maintaining threshold cell densities above critical levels for community assembly; nutrient supplementation strategies (nitrogen, phosphorus, trace elements) supporting initial growth without enriching indigenous competitors; biosurfactant addition improving PFAS bioavailability in sorbed fractions. Integration with engineered treatment systems (constructed wetlands for agricultural runoff, permeable reactive barriers for groundwater plumes, activated sludge augmentation for municipal wastewater) could substantially enhance removal efficiencies while reducing operational costs compared to purely physicochemical approaches (activated carbon adsorption, advanced oxidation, incineration) that generate hazardous secondary waste requiring disposal.

The demonstrated capability for simultaneous multipollutant degradation addresses critical need for complex contaminated sites where mixtures of structurally diverse chemicals require comprehensive treatment. Traditional remediation approaches optimize for single contaminants, achieving high removal for target analyte but leaving cocontaminants untreated (e.g., PFAS-focused activated carbon adsorption does not remove atrazine or lignin; atrazine-targeted biodegradation does not address PFAS). GENIA-designed communities with broad metabolic capacity can potentially serve as ″universal degraders″ for complex contamination scenarios, reducing need for multistage treatment trains and associated costs.

Mechanistically, several questions merit further investigation:^1^ Does consortium adaptation occur over extended time scales (months-years), with horizontal gene transfer between members or mutation-selection dynamics enhancing degradation capacity beyond initial performance?^2^ Can computational predictions guide directed evolution strategies, identifying specific enzyme variants or pathway optimizations most likely to improve consortium function?^3^ How do environmental stressors (temperature extremes, desiccation, competing pollutants, predation by protozoa) affect community stability and function, and can GENIA framework incorporate stress response predictions to design robust communities for harsh environments?^4^ What is minimum inoculation density for successful bioaugmentation, and how does this depend on indigenous microbial competition and environmental conditions?

The demonstration of simultaneous multipollutant degradation addresses critical gaps in environmental biotechnology, as contaminated sites often contain complex pollutant mixtures that require costly sequential treatments.^101^ As regulatory pressure mounts for comprehensive environmental remediation, particularly for persistent pollutants such as PFAS,^8^ engineered communities with defined functional capabilities become increasingly valuable for both remediation and prevention strategies. The framework’s modularity enables expansion to additional pollutant classes, providing a scalable platform for addressing emerging contaminant challenges.^93^

### Broader Implications for Synthetic Ecology

GENIA establishes a proof of concept that machine-learning-guided biological system design can predict and engineer microbial consortia that exhibit emergent properties exceeding those of individual strains or natural communities. This paradigm shift from descriptive ecology (observing communities and inferring principles post hoc) to predictive engineering (designing communities and validating predictions experimentally) presents transformative opportunities across synthetic biology, microbiome engineering, and biotechnology. The workflow’s modularity enables application beyond bioremediation: plant growth promotion (designing rhizosphere consortia enhancing nutrient acquisition, drought tolerance, pathogen resistance), bioprocessing (optimizing mixed cultures for biofuel/biochemical production with division of labor and reduced competition), human/animal microbiome therapeutics (rational design of probiotic consortia treating dysbiosis-associated diseases), and even astrobiology (engineering life support systems for space missions or extraterrestrial colonization).

The integration of high-throughput cultivation, systems genomics, metabolic modeling, and artificial intelligence establishes comprehensive pipeline from environmental isolation through computational design to validated functional product. As regulatory frameworks evolve to accommodate the ecological release of engineered microorganisms (currently restricted but increasingly considered for bioaugmentation applications), rationally designed synthetic communities may become standard tools for environmental management, displacing ad hoc application of undefined enrichments or single strains with unpredictable efficacy.

The current achievement, >90% removal of three persistent pollutants within 7 days and stable community composition, demonstrates soil performance and represents a milestone in synthetic ecology, validating that engineered communities can match or exceed natural systems while providing the predictability and reproducibility essential for practical applications. As training data sets grow (with additional strains sequenced, more phenotypic measurements collected, and more diverse environmental validations performed), machine learning models will improve through network effects, potentially achieving predictive accuracy comparable to that of rational enzyme engineering or metabolic pathway optimization. The GENIA framework establishes a proof of concept for machine-learning-guided design of synthetic microbial communities, demonstrating for the first time that computational approaches can predict and engineer biological systems for complex environmental applications. As machine learning continues to revolutionize biological sciences, our approach exemplifies how artificial intelligence can capture biological intuition to design functional microbial consortia that possess emergent properties exceeding those of individual strains or natural communities, opening new avenues for addressing global environmental challenges through engineered biological solutions.

## Supplementary Material





## Data Availability

All 45 whole-genome sequences were deposited in NCBI GenBank under BioProject PRJNA1370521. Raw sequencing reads in SRA. Full-length 16S rRNA sequencing data in SRA. Complete GENIA framework source code, including Graph Attention Network implementation, Node2Vec integration, and analysis scripts at https://github.com/EsauDelaVega/GENIA_Framework_Biodegradation under MIT license. The repository includes preprocessed genomic features, trained model weights, Jupyter notebooks, comprehensive documentation, and sample data sets.

## Notes

Detailed experimental data, computational analyses, and model validation metrics supporting the main manuscript findings: (Figures S1 and S2) community dynamics and machine learning model performance metrics; (Figure S3) SHAP-based feature importance analysis of the GENIA framework; (Figure S4) model predictions at the individual strain level across all 135 strain-pollutant combinations; (Figure S5) graph attention network architecture and learned attention weights (PDF)
(Tables S1–S6) Experimental data, including strain characteristics, enzyme distributions, degradation performance, kinetic parameters, community composition dynamics, and soil microcosm results; (Tables S7–S14) comprehensive machine learning methodology documentation, including model architecture, hyperparameters, cross-validation metrics, feature importance rankings, Node2Vec embeddings, attention weight statistics, and comparative model benchmarking (XLSX)
