# Supplementary material for: Machine Learning-Guided Synthetic Microbial Communities Enable Functional and Sustainable Degradation of Persistent Environmental Pollutants
Source: Environ Sci Technol. 2026 Apr 27;60(18):13500–19. doi: 10.1021/acs.est.6c01112 (PMC13173533; doi:10.1021/acs.est.6c01112)
Supplement: Supplementary file 1 [file es6c01112_si_001.pdf]

Machine Learning-Guided Synthetic Microbial Communities Enable Functional and Sustainable Degradation of Persistent Environmental Pollutants

Esaú De la Vega-Camarillo[1*], Jorge Arreola-Vargas[1], Sanjay Antony-Babu[1], Saurav Kumar Mathur[1], Joshua Andrew Santos[1], Won Bo Shim[1*]

[1]Department of Plant Pathology and Microbiology, Texas A&M University, College Station, Texas 77843, USA

**Co-corresponding authors: es.delavegacamarillo@ag.tamu.edu* and *wbshim@tamu.edu*

Supplementary Figures

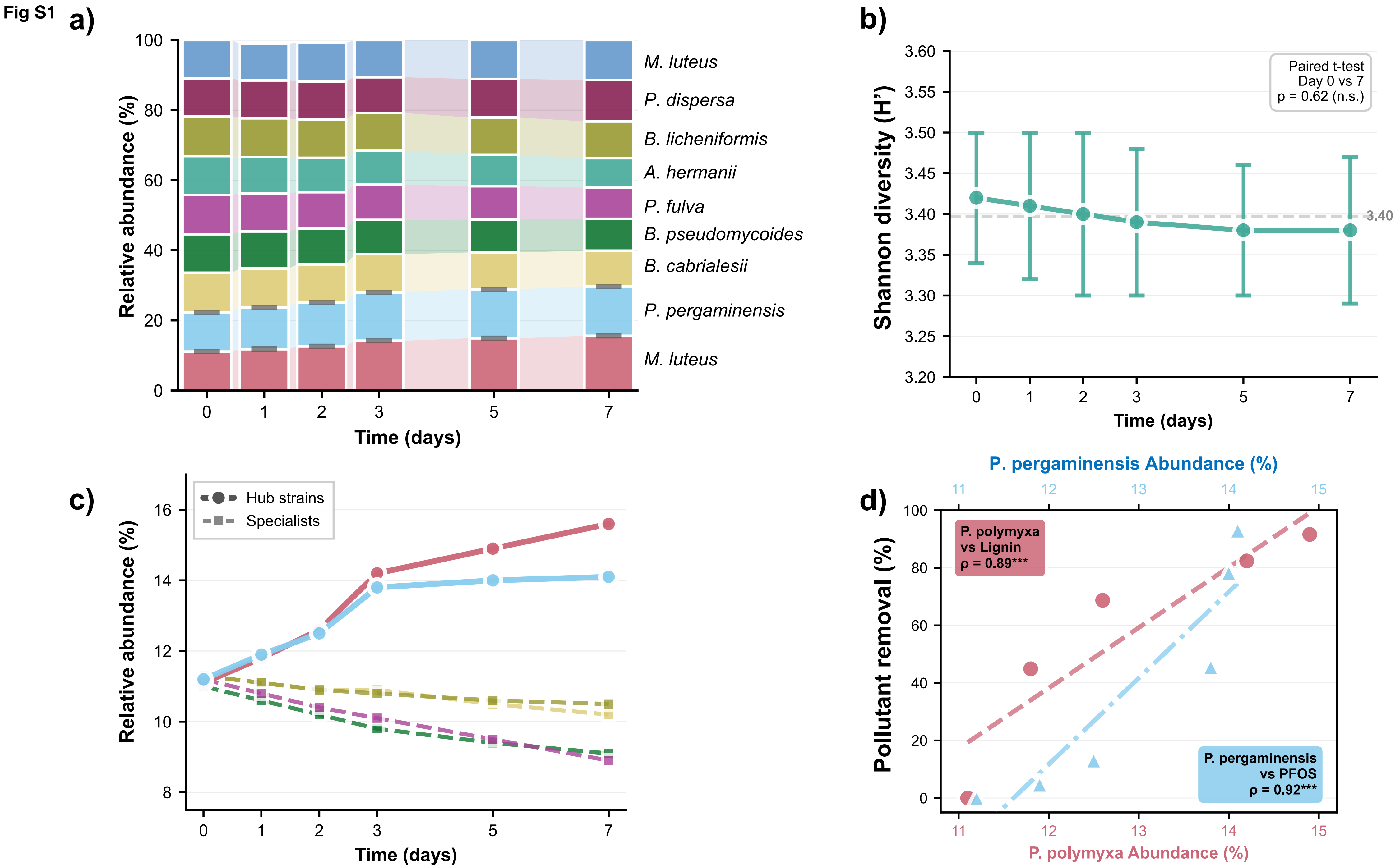

Fig S1
a)
Relative abundance (%)
Time (days)
M. luteus
P. dispersa
B. licheniformis
A. hermanii
P. fulva
B. pseudomycoides
B. cabrialesii
P. pergaminensis
M. luteus
b)
Shannon diversity (H')
Time (days)
Paired t-test
Day 0 vs 7
p = 0.62 (n.s.)
3.40
c)
Relative abundance (%)
Time (days)
Hub strains
Specialists
d)
P. pergaminensis Abundance (%)
Pollutant removal (%)
P. polymyxa vs Lignin ρ = 0.89***
P. pergaminensis vs PFOS ρ = 0.92***
P. polymyxa Abundance (%)

Fig S2

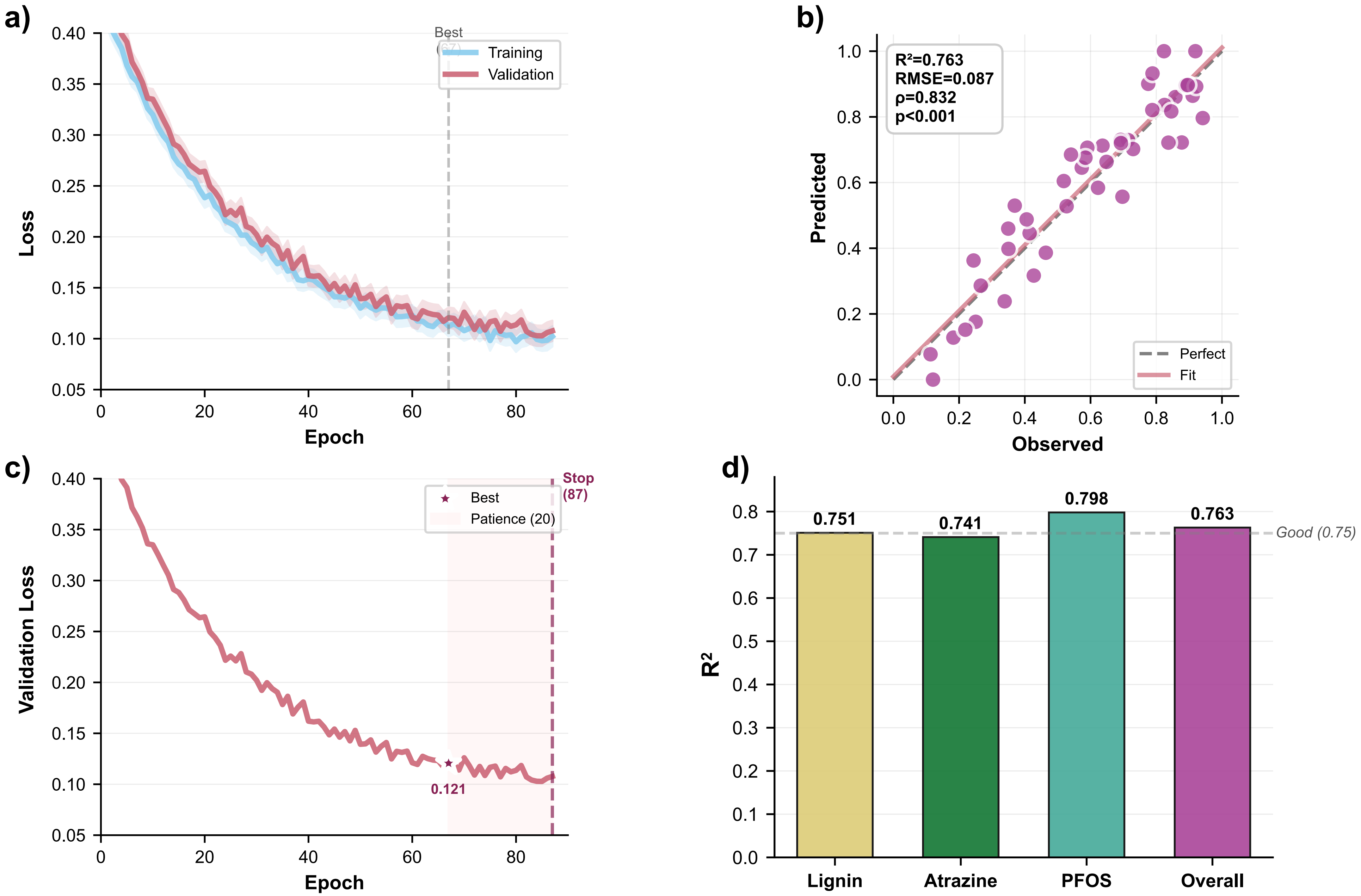
a)
Best
(67)
Training
Validation
Loss
Epoch
b)
R²=0.763
RMSE=0.087
ρ=0.832
p<0.001
Predicted
Observed
Perfect
Fit
c)
Stop
(87)
Best
Patience (20)
0.121
Validation Loss
Epoch
d)
0.751
0.741
0.798
0.763
Good (0.75)
R²
Lignin
Atrazine
PFOS
Overall

Fig S3

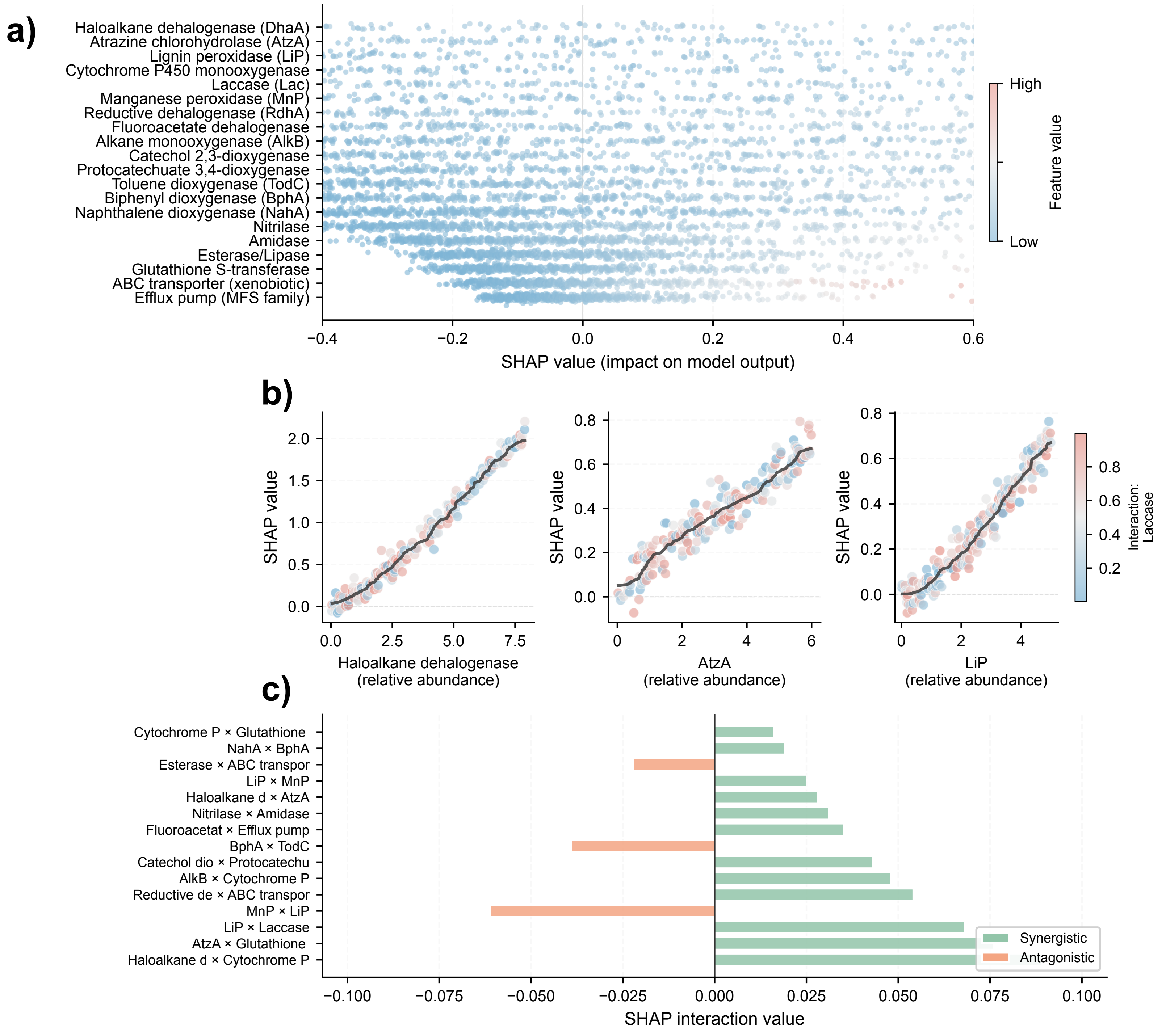
a)
Haloalkane dehalogenase (DhaA)
Atrazine chlorohydrolase (AtzA)
Lignin peroxidase (LiP)
Cytochrome P450 monooxygenase
Laccase (Lac)
Manganese peroxidase (MnP)
Reductive dehalogenase (RdhA)
Fluoroacetate dehalogenase
Alkane monooxygenase (AlkB)
Catechol 2,3-dioxygenase
Protocatechuate 3,4-dioxygenase
Toluene dioxygenase (TodC)
Biphenyl dioxygenase (BphA)
Naphthalene dioxygenase (NahA)
Nitrilase
Amidase
Esterase/Lipase
Glutathione S-transferase
ABC transporter (xenobiotic)
Efflux pump (MFS family)
High
Feature value
Low
−0.4
−0.2
0.0
0.2
0.4
0.6
SHAP value (impact on model output)
b)
SHAP value
Haloalkane dehalogenase
(relative abundance)
AtzA
(relative abundance)
LiP
(relative abundance)
Interaction:
Laccase
c)
Cytochrome P × Glutathione
NahA × BphA
Esterase × ABC transpor
LiP × MnP
Haloalkane d × AtzA
Nitrilase × Amidase
Fluoroacetat × Efflux pump
BphA × TodC
Catechol dio × Protocatechu
AlkB × Cytochrome P
Reductive de × ABC transpor
MnP × LiP
LiP × Laccase
AtzA × Glutathione
Haloalkane d × Cytochrome P
Synergistic
Antagonistic
−0.100
−0.075
−0.050
−0.025
0.000
0.025
0.050
0.075
0.100
SHAP interaction value

Fig S4

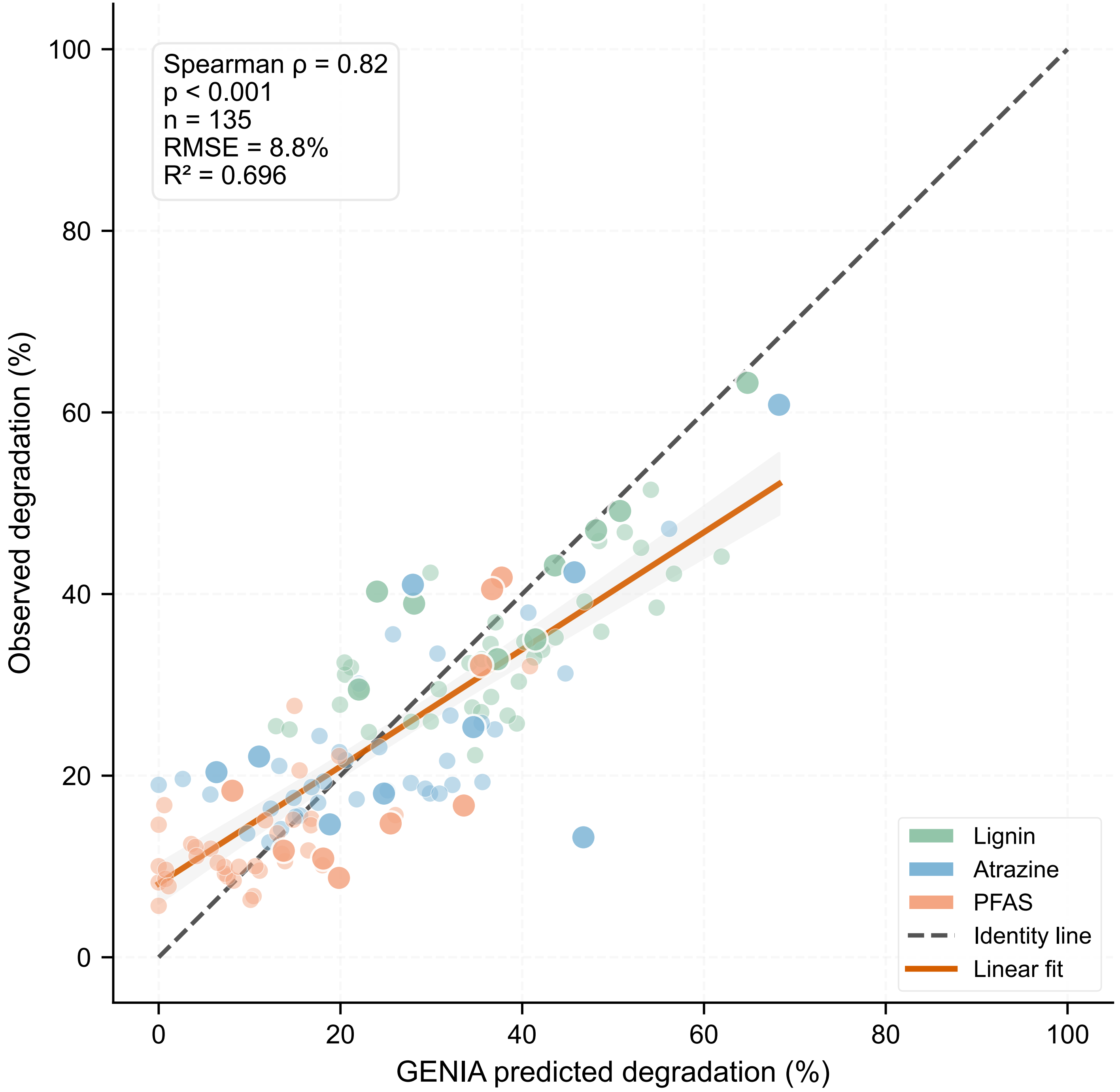

Spearman ρ = 0.82
p < 0.001
n = 135
RMSE = 8.8%
R² = 0.696
Observed degradation (%)
GENIA predicted degradation (%)
0
20
40
60
80
100
Lignin
Atrazine
PFAS
Identity line
Linear fit

Fig S5

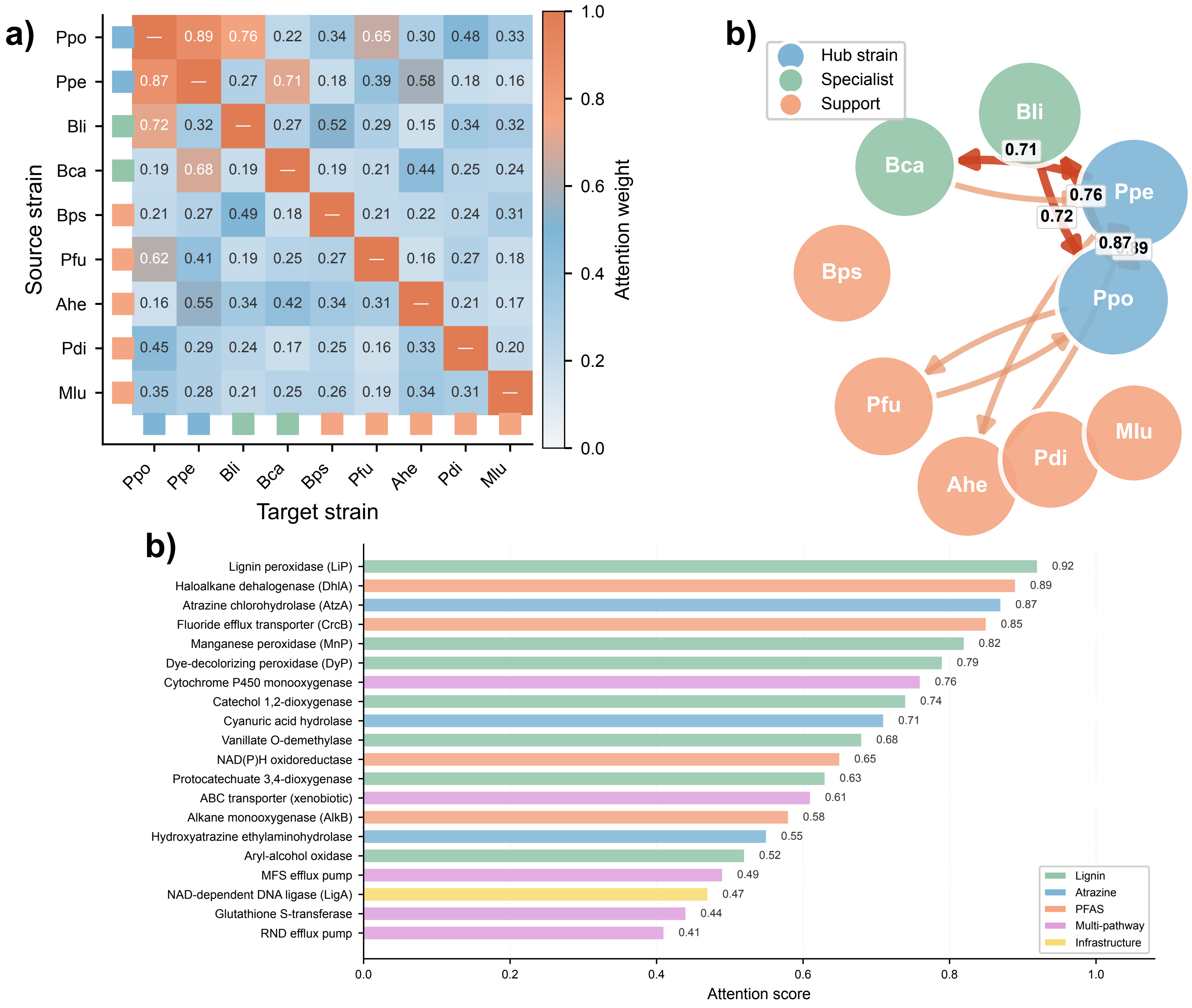

a)
Source strain
Target strain
Attention weight
1.0
0.8
0.6
0.4
0.2
0.0
Ppo — 0.89 0.76 0.22 0.34 0.65 0.30 0.48 0.33
Ppe 0.87 — 0.27 0.71 0.18 0.39 0.58 0.18 0.16
Bli 0.72 0.32 — 0.27 0.52 0.29 0.15 0.34 0.32
Bca 0.19 0.68 0.19 — 0.19 0.21 0.44 0.25 0.24
Bps 0.21 0.27 0.49 0.18 — 0.21 0.22 0.24 0.31
Pfu 0.62 0.41 0.19 0.25 0.27 — 0.16 0.27 0.18
Ahe 0.16 0.55 0.34 0.42 0.34 0.31 — 0.21 0.17
Pdi 0.45 0.29 0.24 0.17 0.25 0.16 0.33 — 0.20
Mlu 0.35 0.28 0.21 0.25 0.26 0.19 0.34 0.31 —
Ppo Ppe Bli Bca Bps Pfu Ahe Pdi Mlu
b)
Hub strain
Specialist
Support
Bli
Bca
Ppe
Bps
Ppo
Pfu
Mlu
Pdi
Ahe
0.71
0.76
0.72
0.87
b)
Lignin peroxidase (LiP) 0.92
Haloalkane dehalogenase (DhlA) 0.89
Atrazine chlorohydrolase (AtzA) 0.87
Fluoride efflux transporter (CrcB) 0.85
Manganese peroxidase (MnP) 0.82
Dye-decolorizing peroxidase (DyP) 0.79
Cytochrome P450 monooxygenase 0.76
Catechol 1,2-dioxygenase 0.74
Cyanuric acid hydrolase 0.71
Vanillate O-demethylase 0.68
NAD(P)H oxidoreductase 0.65
Protocatechuate 3,4-dioxygenase 0.63
ABC transporter (xenobiotic) 0.61
Alkane monooxygenase (AlkB) 0.58
Hydroxyatrazine ethylaminohydrolase 0.55
Aryl-alcohol oxidase 0.52
MFS efflux pump 0.49
NAD-dependent DNA ligase (LigA) 0.47
Glutathione S-transferase 0.44
RND efflux pump 0.41
Lignin
Atrazine
PFAS
Multi-pathway
Infrastructure
0.0
0.2
0.4
0.6
0.8
1.0
Attention score
